# Enhancing the antimicrobial activity of silver nanoparticles against ESKAPE bacteria and emerging fungal pathogens by using tea extracts†

**DOI:** 10.1039/d3na00220a

**Published:** 2023-08-23

**Authors:** Sada Raza, Mateusz Wdowiak, Mateusz Grotek, Witold Adamkiewicz, Kostiantyn Nikiforow, Pumza Mente, Jan Paczesny

**Affiliations:** a Institute of Physical Chemistry, Polish Academy of Sciences Kasprzaka 44/52 01-224 Warsaw Poland jpaczesny@ichf.edu.pl +48 22 343 2071; b Military University of Technology gen. Sylwestra Kaliskiego 2 00-908 Warsaw Poland

## Abstract

The sale of antibiotics and antifungals has skyrocketed since 2020. The increasing threat of pathogens like ESKAPE bacteria (*Enterococcus faecium*, *Staphylococcus aureus*, *Klebsiella pneumoniae*, *Acinetobacter baumannii*, *Pseudomonas aeruginosa*, and *Enterobacter* spp.), which are effective in evading existing antibiotics, and yeasts like *Candida auris* or *Cryptococcus neoformans* is pressing to develop efficient antimicrobial alternatives. Nanoparticles, especially silver nanoparticles (AgNPs), are believed to be promising candidates to supplement or even replace antibiotics in some applications. Here, we propose a way to increase the antimicrobial efficiency of silver nanoparticles by using tea extracts (black, green, or red) for their synthesis. This allows for using lower concentrations of nanoparticles and obtaining the antimicrobial effect in a short time. We found that AgNPs synthesized using green tea extract (G-TeaNPs) are the most effective, causing approximately 80% bacterial cell death in Gram-negative bacteria within only 3 hours at a concentration of 0.1 mg mL^−1^, which is better than antibiotics. Ampicillin at the same concentration (0.1 mg mL^−1^) and within the same duration (3 h) causes only up to 40% decrease in the number of *S. aureus* and *E. cloacae* cells (non-resistant strains). The tested silver nanoparticles also have antifungal properties and are effective against *C. auris* and *C. neoformans*, which are difficult to eradicate using other means. We established that silver nanoparticles synthesized with tea extracts have higher antibacterial properties than silver nanoparticles alone. Such formulations using inexpensive tea extracts and lower concentrations of silver nanoparticles show a promising solution to fight various pathogens.

## Abstract 2

The sale of antibiotics and antifungals has skyrocketed since 2020.

## Graphical abstract

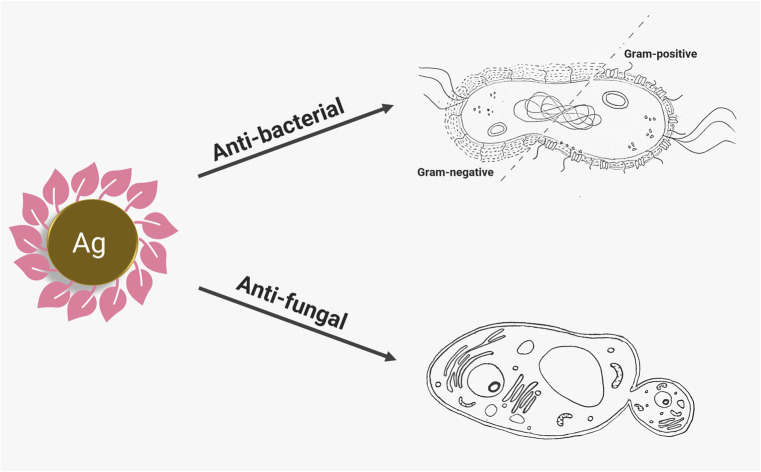

## Introduction

The fight against drug-resistant microorganisms costs the US alone $55 billion every year. $20 billion is spent on health care, and $35 billion is due to the loss of productivity.^1^ The overuse of antibiotics in medicine^2^ and agriculture (to stimulate the growth of livestock^3^) resulted in the antimicrobial resistance (AMR) epidemic. Since 2015, it has been estimated that about 154 million prescriptions of antibiotics are reported yearly; about 30% of these prescriptions are considered unnecessary.^4^ Since 2016, AMR has been considered by the United Nations as a threat to health and human development.^5^ Bacteria can develop drug-resistance mechanisms within 10 hours of antibiotic exposure.^6^ Drug resistance can also be transmitted between bacterial species *via* different means, including horizontal gene transfer of antibiotic resistance genes (ARGs).^7^

The spread of antibiotic resistance resulted in the appearance of multi-drug-resistant (resistant to more than three classes of antibiotics) bacterial strains. In the case of Gram-negative bacteria, the spread of extended-spectrum beta-lactamase (ESBL)-positive strains made the usage of beta-lactam antibiotics (*e.g.*, penicillin, cephalosporins and monobactams) less effective.^8^ Among Gram-positive bacteria, beta-lactam antibiotics resistance is usually due to the development of bacterial penicillin-binding proteins (PBPs).^9–11^ PBPs bind to all beta-lactam antibiotics.^12^ MDR infections can still be treated with drugs such as carbapenems (against Gram-negative bacteria) and vancomycin (against Gram-positive bacteria). However, some bacterial groups already resist these ‘last-chance antibiotics’.^13–16^*Enterococci* species (*Enterococcus faecium*, and *Enterococcus faecalis*) are generally known for their natural-borne resistance to vancomycin.^17^ The main reservoir of carbapenem resistance (CR), acquired due to the production of carbapenemases, is *Klebsiella pneumoniae*^18–20^ and *Acinetobacter baumannii*^21,22^ strains. These most widespread and problematic bacterial pathogens were grouped and described under the ESKAPE acronym. ESKAPE bacteria are *E. faecium*, *Staphylococcus aureus*, *K. pneumoniae*, *A. baumannii*, *Pseudomonas aeruginosa*, and *Enterobacter* spp. These are opportunistic pathogens that are responsible for numerous nosocomial infections and the spread of AMR worldwide.^23,24^

Although bacterial pathogens are a severe threat, as their infections may lead to sepsis,^25^ various cancers,^26^ and metabolic disorders,^27^ the problem of emerging pathogens is far beyond just bacteria. Drug-resistant fungi (mainly yeasts) became a new threat pertaining to nosocomial infection. Cryptococcosis, an infection caused by the yeast *Cryptococcus neoformans*, has been a significant problem for immunodeficient patients for years.^28^ The therapy is challenging because cerebral infection is the most frequent form of cryptococcosis.^29^ Moreover, azole drugs such as voriconazole are frequently used for severe cryptococcosis because a significantly lower dose (approximately seven times lower) can be used compared to that of amphotericin B.^30^ However, 11–19% of patients exposed to voriconazole were found to have elevated aminotransferase levels.^31^

Another emerging fungal pathogen is *Candida auris*. First discovered in 2009, *C. auris* has been detected within a few years in different geological regions, including wealthy counties, *e.g.*, South Korea, Canada, and the USA.^32^ It has been reported to cause fungemia (the presence of fungal cells in the bloodstream),^33^ which is particularly dangerous for immunosuppressed patients, children, and elders.^34^ In a murine model, the fatality of *C. auris* infection is comparable to that of *Candida albicans*, *Candida haemulonii*, and *Candida glabrata* (survival rate of about 30% within 15 days post-infection).^35^ The average mortality rate for infected humans is about 35%,^36^ comparable to the fatality of *Candida* spp. infection in immunodeficient patients.^37^ Moreover, *C. auris* is also resistant to commonly used antifungal drugs, such as amphotericin B, fluconazole, voriconazole, or echinocandin.^38–40^ The isolation of pan-resistant *C. auris* strains (resistant to four groups of antifungal drugs) has been already reported.^8,11,15^ Combined with the unknown mechanism of rapid gain of fungistatic resistance,^36,41^ its potential spread to *Cryptococcus* spp. and *Candida* spp. may become a significant medical problem in the near future.

New biomedical applications have emerged with the growing advancements in nanotechnology linked with microbiology, especially with silver nanoparticles (AgNPs). Due to the broad spectrum of antibacterial, antifungal, and antiviral properties, AgNPs are popularly adopted as disinfectants and antimicrobial agents.^42^ More than 500 tons of nanoparticles per year are now supplied to meet different industrial demands (with the perspective to reach the amount of 1230 tons per year),^43^ drawing attention to their biological activity and safety along with their mechanism of action.^44^ Silver nanoparticles cause microbial cell death by penetrating bacterial cell walls, altering the cell membrane structure, increasing cell membrane permeability, producing reactive oxygen species (ROS), and interrupting the replication of DNA.^42^ More than a 3 log reduction in the CFU per mL (colony forming units per milliliter) of Gram-negative bacteria was witnessed in the presence of silver nanoparticles by impairing their adhesion ability and preventing biofilm formation.^45,46^ AgNPs are generally considered safe for humans.^47^ However, due to their potential toxicity to aquatic environments^48^ and the possibility of bacterial resistance to nanoparticles,^49^ they may generate problems soon.

Applying natural extracts with antibacterial properties appears to be a promising approach to support nanotechnology-based solutions. Among the products from different plants, the extracts of *Camellia sinensis* (tea) are obvious choices. Tea and tea extracts have antioxidant and antibacterial properties due to the containment of numerous flavanols and polyphenols.^50,51^ Polyphenols in tea extracts (especially catechins) act as capping and reducing agents.^52,53^ In the presence of metal ions, the hydroxyl groups of catechin molecules act as electron donors, reducing silver ions (Ag^+^) to metallic silver (Ag^0^).^54^ Surface interactions between polyphenols and newly reduced metallic silver particles function as the capping factor preventing the agglomeration of AgNPs.^53^ Combining silver nanoparticles and tea extracts against microbes has drawn attention within the past few years.^50,55,56^ The antimicrobial activity of green-synthesized AgNPs (including natural extracts) is described in the recent review by Xin *et al.*^57^

In this paper, we propose a method to reduce the working concentration of AgNPs, owing to their synthesis with natural extracts. We synthesized AgNPs with natural extracts from three different types of tea – black, green, and red (B-TeaNPs, G-TeaNPs, and R-TeaNPs) and tested them against multidrug-resistant pathogenic bacteria and yeasts. Their antimicrobial properties were compared in selected concentrations (between 0.1 mg mL^−1^ to 1 mg mL^−1^). The strains chosen for antibacterial tests were the representative strains of ESKAPE bacteria – *E. faecium*, *S. aureus*, *K. pneumoniae*, *A. baumannii*, *P. aeruginosa*, and *Enterobacter cloacae*. Antifungal properties of TeaNPs were examined on *C. auris* and *C. neoformans*. This study is the first to investigate and compare the antimicrobial properties of different sets of TeaNPs on such a wide range of pathogenic microbes (including ESKAPE bacteria and fungi).

## Experimental

### Synthesis of AgNPs

All commercially available reagents were used as received without further purification. According to the product specifications provided by the producer (Sigma Aldrich), the purity of reagents was >98%, estimated using GC analysis or titration with appropriate reagents.

A control batch of silver nanoparticles was prepared following the method described by Agnihotri *et al.* with slight modifications.^58^ An aqueous solution (48 mL) containing 2 mM NaBH_4_ and 2 mM trisodium citrate (TSC) was heated to 60 °C and stirred for 30 minutes. Next, the AgNO_3_ solution (2 mL, 11.7 mM) was added dropwise. The mixture was heated to 90 °C, and the pH was adjusted to 10.5 using 0.1 M NaOH. Finally, the reaction was stirred further at 90 °C for 20 minutes until an evident color change was observed.

### Synthesis of silver nanoparticles with tea extracts

Silver nanoparticles with tea extracts (TeaNPs) were synthesized using AgNO_3_ and tea extracts by a slightly modified method by Nakhjavani *et al.*^59^ Three kinds of tea: black (Loyd, Mokate, and Poland), green (Loyd, Mokate, and Poland), and Pu-erh (Lord Nelson, Lidl, and Poland), were frozen in liquid nitrogen and ground using a mortar and pestle. 10 g of the ground leaves were immersed in hot water (60 °C, 100 mL) and left to brew. After 15 minutes of steeping, the raw extracts were cooled to room temperature, transferred to falcon tubes, and centrifuged (9000 rpm, 10 min) to remove leaf residuals. Decanted liquids were centrifuged again (15 000 rpm, 10 min) to remove fine debris. Finally, extracts were filtered with a syringe filter (pore size 0.22 nm). After this, a portion (25 mL) of the selected extract was transferred into an Erlenmeyer flask, and AgNO_3_ (750 mL, 10 mM) was added dropwise while stirring, maintaining a constant temperature below 50 °C. The solution was allowed to mix for 2 hours, during which the color of the reaction mixture changed to yellow-brown with a silver shine. The resulting nanoparticle suspensions were centrifuged (10 000 rpm, 10 min). The supernatant was discarded, and the deposit was redispersed in water by sonication. The purification process was repeated 6 times, and finally, the nanoparticles were suspended in water (resulting concentration of 1 mg mL^−1^, estimated by drying the nanoparticles and further dilution), creating a stock suspension, which was stored in a fridge (4 °C) for later use.

### Characterization – protocols and equipment

UV-vis absorption spectra were recorded using a Thermo Scientific Evolution 220 spectrometer with a temperature controller. The measurements were carried out using 1 cm quartz cuvettes (Hellma, Germany).

Fourier-transform infrared spectroscopy (FTIR) studies were performed with a Vertex 80 V FTIR spectrometer (Bruker, USA) equipped with a Platinum ATR (Bruker, USA) module. The tea extracts were dried by rotary evaporation at 65 °C to remove water. The TeaNPs were centrifuged to remove water and then dried in an oven at 65 °C. Dried powder samples were placed on a diamond prism (1 mm × 1 mm) to cover it. The spectral resolution of the measurement was 2 cm^−1^, and the number of scans was 64.

The hydrodynamic sizes of the NPs were determined using the dynamic light scattering (DLS) technique. The DLS measurements were conducted with a Malvern ZetaSizer Nano-ZS instrument using 1 cm quartz cuvettes (Hellma, Germany).

Scanning electron microscopy (SEM) images were collected with a Nova NanoSEM 450 under high vacuum (10^−7^ mbar). The purified samples were deposited on a silicon substrate, allowed to dry, and mounted onto a standard SEM stub with carbon tape. The images were collected using a through lens detector (TLD) of secondary electrons at a primary beam energy of 10 kV and a 4.8 mm working distance. The average diameters of the NPs were calculated from the SEM images using the ImageJ software by measuring at least 100 particles per sample.

High-resolution transmission electron microscopy (HRTEM) was performed using an FEI TECNAI G2 F20S-TWIN microscope. 10 μL of liquid samples of TeaNP stock solutions were placed on Quantifoil Cu 300 holey carbon meshes and left to dry.

X-ray diffraction analysis was performed with a Malvern PANalytical Empyrean range diffractometer at room temperature with the wavelength *λ* = 0.154 nm.

X-ray photoelectron spectroscopy (XPS) was performed with a CLAM2 XPS spectrometer (VG Microtech Ltd, London, United Kingdom). Dried powder samples were placed on the measurement table to cover the appropriate surface. Measurement was performed in a vacuum, within the binding energy ranging from 0–1300 eV.

The surface areas of tested materials were measured with an ASAP 2020 automatic analyzer (Micromeritics Instrument Corp., USA), using krypton as an adsorbate. Before adsorption measurements at liquid nitrogen temperature (77 K), the samples were outgassed at 373 K for 15 hours in a vacuum chamber to clean their surface. The specific surface areas of AgNPs were calculated using the BET (Brunauer–Emmett–Teller) method.

### Bacteria

For the evaluation of the antibacterial properties of TeaNPs, *E. faecium* DSM 13590 (obtained from the German Collection of Microorganisms and Cell Cultures), *S. aureus* DSM 105272 (obtained from the German Collection of Microorganisms and Cell Cultures), *A. baumannii* ATCC 19606 (obtained from the collection of the Institute of Physical Chemistry PAS), *K. pneumoniae* ATCC 700603 (obtained from the collection of the Institute of Physical Chemistry PAS), *P. aeruginosa* DSM24068 (obtained from the German Collection of Microorganisms and Cell Cultures), *E. cloacae* PCM 2569 (obtained from the Polish Collection of Microorganisms), and *Escherichia coli* BL21 (obtained from the Institute of Biochemistry and Biophysics PAS, Warsaw, Poland) were used. A colony of the required strain was picked from the stock plates and transferred to 10 mL of LB medium to prepare the overnight bacterial cultures (37 °C, 200 rpm, using an orbital shaker-incubator ES-20). The overnight cultures were refreshed by adding fresh LB medium (1 : 4 v/v) and incubated at 37 °C for approximately 1 hour. We aimed to reach the appropriate OD_600_ corresponding to the known concentration of bacteria expressed as CFU per mL (for *E. faecium* OD_600_ ∼ 0.5 ≥ 1.0 × 10^7^ CFU per mL; *S. aureus* OD_600_ ∼ 1.0 ≥ 1.5 × 10^9^ CFU per mL; *K. pneumoniae* OD_600_ = 1.0 ≥ 1.0 × 10^8^ CFU per mL; *A. baumannii* OD_600_ = 1.0 ≥ ∼4.0 × 10^8^ CFU per mL; *P. aeruginosa* OD_600_ = 1.0 ≥ ∼5.0 × 10^9^ CFU per mL; *E. cloacae* OD_600_ = 0.5 ≥ 1.0 × 10^9^ CFU per mL; *E. coli* OD_600_ = 1.0 ≥ 1.0 × 10^8^ CFU per mL). Each bacterial culture was diluted in 0.9% NaCl to an initial concentration of about 10^4^ cells per mL. The mixtures containing bacterial cultures and TeaNPs at the concentrations of 1.0 mg mL^−1^, 0.5 mg mL^−1^, or 0.1 mg mL^−1^ were plated on the LB agar plates (100 μL per plate), and then incubated with shaking (room temperature, 220 rpm) for 3 hours. Control samples did not contain any AgNPs. After the incubation, another 100 μL of each suspension was cultured onto the fresh LB agar plates. The plates were incubated overnight at 37 °C, and then the number of bacteria was calculated based on the colony number, according to the equation CFU per mL = *N* × *D* × 10 (*N* – number of colonies; *D* – dilution). Student's *t*-test was performed to evaluate whether observed differences, compared to the adequate control, were statistically significant (*p < 0.05; **p < 0.01; ***p < 0.001). The experiments were conducted in triplicate.

### Antibiotic and tea extract assay

To evaluate the antibacterial properties of ampicillin (Sigma Aldrich), *S. aureus* DSM 105272 and *E. cloacae* PCM 2569 were used. A colony of the required strain was picked from the stock plates and transferred to 10 mL of LB medium to prepare the overnight bacterial cultures (incubated at 37 °C in an orbital shaker-incubator ES-20, 200 rpm). The overnight cultures were refreshed by adding fresh LB medium (1 : 4 v/v) and incubated at 37 °C for approximately 1 hour. We aimed to reach the appropriate OD_600_ corresponding to the known concentration of bacteria expressed as CFU per mL (for *S. aureus* OD_600_ ∼ 1.0 ≥ 1.5 × 10^9^ CFU per mL; for *E. cloacae* OD_600_ = 0.5 ≥ 1.0 × 10^9^ CFU per mL). Each bacterial culture was diluted in 0.9% NaCl to an initial concentration of about 10^4^ cells per mL. The mixtures contain bacterial cultures and ampicillin at concentrations of 0.01 mg mL^−1^ (MIC value for ampicillin) and 100 μg mL^−1^ (0.1 mg mL^−1^). Tea extracts, obtained according to the protocol described in the *Synthesis of silver nanoparticles with tea extracts* section, were dried using a rotatory evaporator (Rotavapor R-100, Buchi AG, Switzerland) and then suspended in 0.9% NaCl. The final concentration of tea extracts was 0.1 mg mL^−1^. Bacteria were incubated with antibiotic or tea extract solutions with shaking (room temperature, 220 rpm) for 3 hours. Control samples contained neither antibiotic nor tea extracts. After 3 hours of incubation, another 100 μL of each suspension was cultured onto the fresh LB agar plates. The plates were incubated overnight at 37 °C, and then the number of bacteria was calculated based on the colony number, according to the equation CFU per mL = *N* × *D* × 10 (*N* – number of colonies; *D* – dilution). Student's *t*-test was performed to evaluate whether observed differences, compared to the adequate control, were statistically significant (*p < 0.05; **p < 0.01; ***p < 0.001). The experiments were conducted in triplicate.

### Yeasts

Colonies of *Candida auris* DSM 105986 (obtained from the German Collection of Microorganisms and Cell Cultures, Braunschweig, Germany; also CDC AR-Bank 0381) and *Cryptococcus neoformans* var. *grubii* ATCC 14116 (obtained from the collection of Pomeranian Medical University in Szczecin) were picked from the stock plates and transferred to 10 mL of YPD medium and incubated at 37 °C in an orbital shaker-incubator ES-20, at 200 rpm. The overnight culture was refreshed by mixing 2.5 mL of overnight yeasts cutlure with 7.5 mL of fresh YPD (1 : 4 v/v) medium and incubating at 37 °C for approximately 1 h to reach the OD_600_ ∼ 1.0 (corresponding to around 1.0 × 10^7^ cells per mL) for both yeast species. Such refreshed cultures were diluted in 0.9% NaCl to an initial concentration of about 10^4^ cells per mL. The mixtures containing yeast cultures and TeaNPs (concentrations of 1.0 mg mL^−1^, 0.5 mg mL^−1^, or 0.1 mg mL^−1^) were plated on the YPD agar plates (100 μL per plate) and incubated with shaking (room temperature, 220 rpm) for 3 hours. Control samples did not contain the TeaNPs. After incubation, another 100 μL of each suspension was cultured onto the fresh YPD agar plates. The plates were incubated at 37 °C for 48 hours (*C. auris*) or 96 hours (*C. neoformans*). The number of yeast cells was calculated based on the colony number, according to the equation cells per mL = *N* × *D* × 10 (*N* – number of colonies; *D* – dilution). The yeasts were also exposed to 0.5 mg mL^−1^ tea extracts, according to the protocol described in the subsection *Antibiotic and tea extracts assay*. Student's *t*-test was performed to evaluate whether observed differences, compared to the adequate control, were statistically significant (*p < 0.05; **p < 0.01; ***p < 0.001). The experiments were conducted in triplicate.

## Results & discussion

### Characterization of nanoparticles

**Fig. 1:**
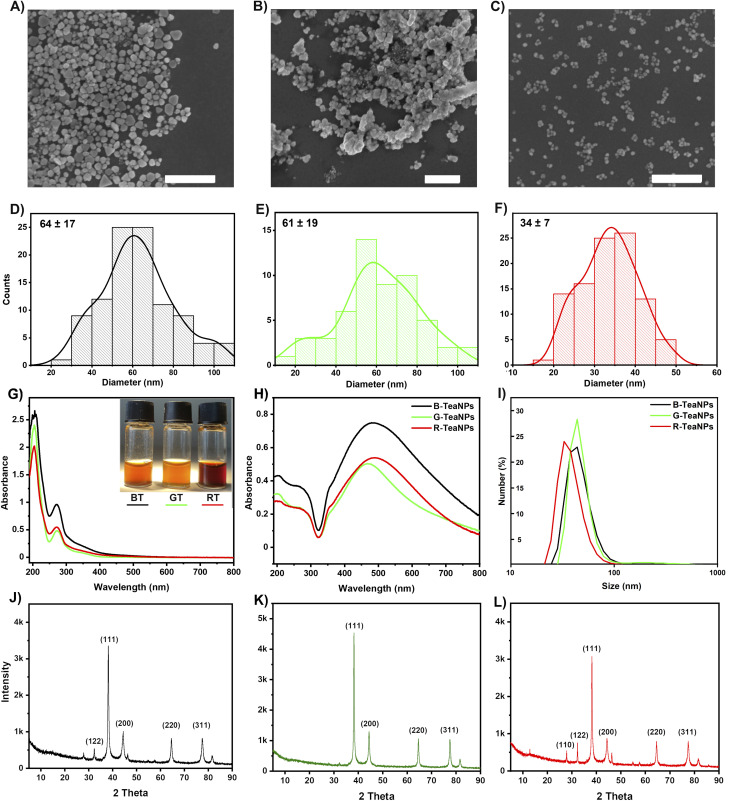
The characterization of the synthesized TeaNPs: SEM pictures of (A) B-TeaNPs, (B) G-TeaNPs, and (C) R-TeaNPs. Scale bars correspond to 500 nm. Size distributions of (D) B-TeaNPs, (E) G-TeaNPs, and (F) R-TeaNPs; (G) UV-vis spectra of tea extracts, (H) UV-vis spectra of TeaNPs, and the spectra were normalized. (I) DLS size estimation of TeaNPs. The XRD diffractograms of (J) B-TeaNPs, (K) G-TeaNPs, and (L) R-TeaNPs.

**Fig. 2:**
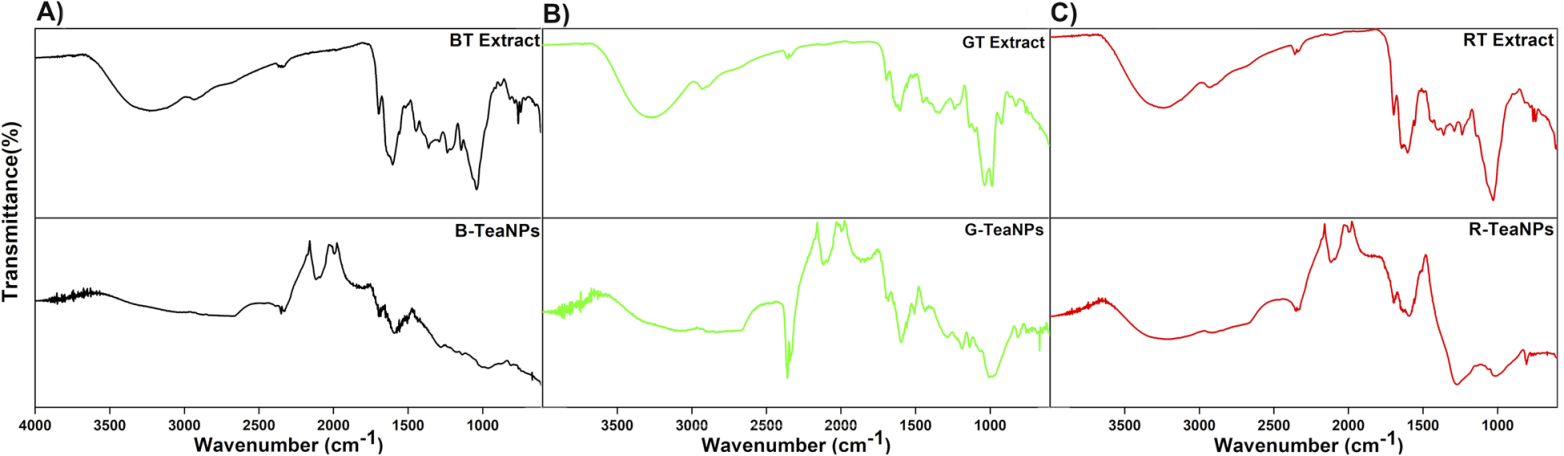
FTIR spectra of (A) black tea extract and B-TeaNPs; (B) green tea extract and G-TeaNPs; (C) red tea extract and R-TeaNPs. The top line on each graph marks 100% transmittance.

**Fig. 3:**
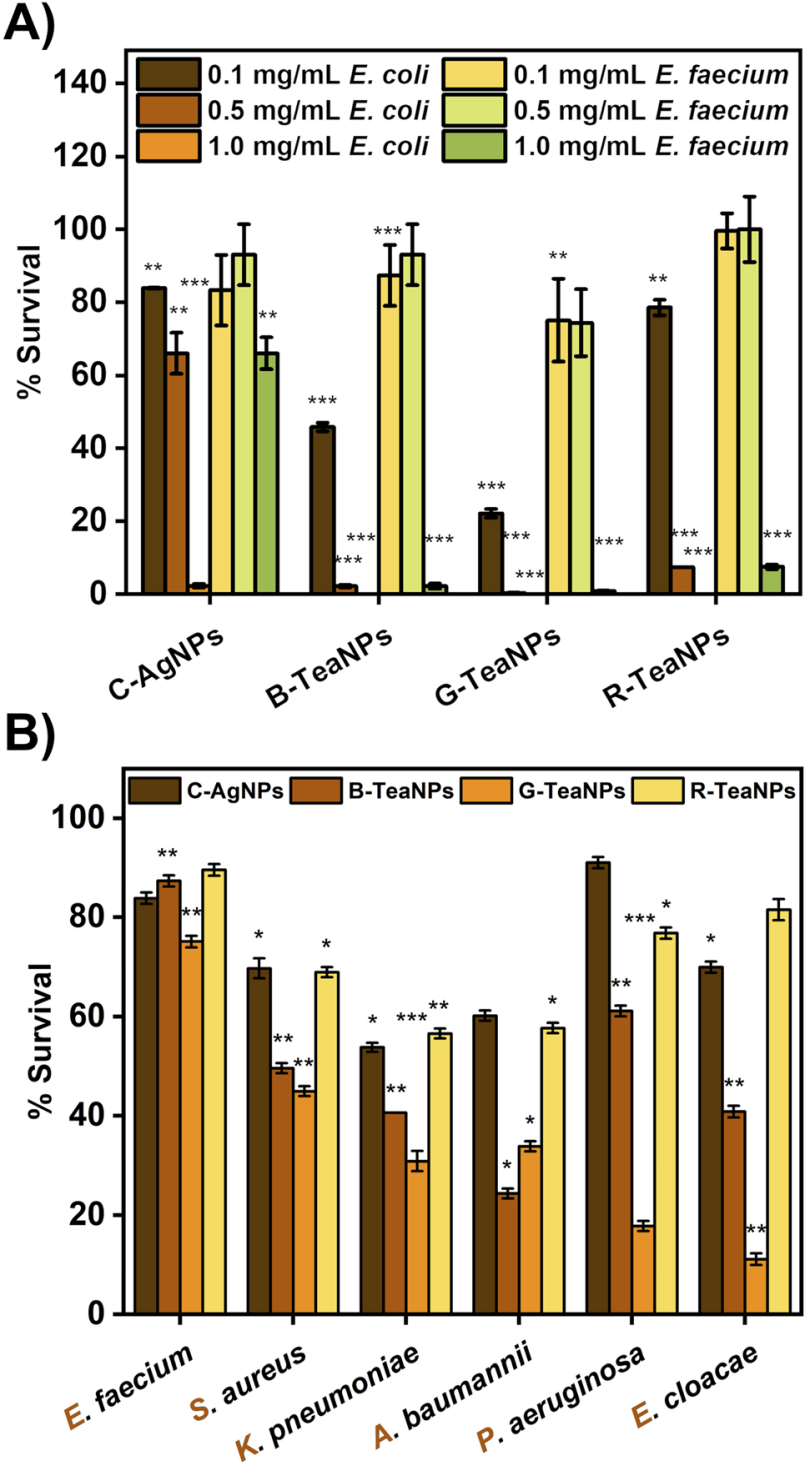
(A) Dose compensation of TeaNPs against Gram-positive (*E. faecium*) and Gram-negative (*E. coli*) bacteria with three concentrations: 0.1 mg mL^−1^, 0.5 mg mL^−1^, and 1 mg mL^−1^. (B) Antibacterial effect of AgNPs (0.1 mg mL^−1^) against the ESKAPE bacterial strains. The results are presented as a percentage of survival (*i.e.*, 
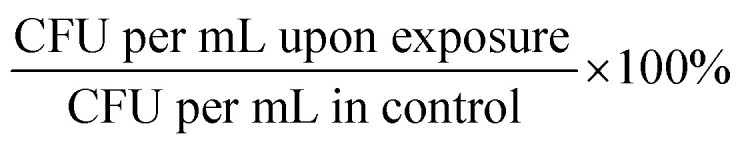
), *p < 0.05; **p < 0.01; ***p < 0.001, *p* values were calculated with respect to the control sample (not exposed to any AgNPs).

We used three popular varieties of tea: black, green, and Pu-erh tea (a type of red tea) to synthesize silver nanoparticles. Components of tea extracts acted as both reducing and capping agents. The characterization of tea extracts and TeaNPs (silver nanoparticles stabilized with tea extracts) was performed employing SEM (Fig. 1A–F), UV-vis spectroscopy (Fig. 1G and H), DLS (Fig. 1I), FTIR (Fig. 2), XRD (Fig. 3), XPS, BET, and HRTEM (Fig. S2 and S3†) techniques. We obtained TeaNPs with cores of average diameters of 64 ± 17 nm (black tea, B-TeaNPs), 61 ± 19 nm (green tea, G-TeaNPs), and 34 ± 7 nm (red tea, R-TeaNPs). B-TeaNPs and G-TeaNPs were polydisperse and irregular in shape (Fig. 1A–C), and in size distribution histograms (Fig. 1D–F). R-TeaNPs were smaller and had a more uniform shape and narrower size distribution. The data from SEM (Fig. 1D–F) matched results obtained using DLS (Fig. 1I). The data on the characterization of control AgNPs (capped with only citrates, C-AgNPs) revealed particles around 30 nm in diameter (SEM) (Fig. S1A†) with a hydrodynamic diameter, measured by DLS, of 28 ± 7 nm (Fig. S1B†).

The UV-vis spectrum of the G-TeaNPs revealed a plasmonic peak of relatively low intensity at 467 nm. B-TeaNPs showed maximum absorption at 485 nm and R-TeaNPs, at 490 nm. This indicated that the R-TeaNPs were bigger than the B-TeaNPs and G-TeaNPs, which was in opposition to SEM measurements. Mock *et al.* showed that the wavelength of the AgNP plasmon resonance peak depends strongly on the particles’ shape.^60^ They showed that for spherical particles of sizes ranging from 40 to 100 nm, a maximum plasmon resonance peak appears at around 410 nm to around 500 nm, for pentagons of size from 60 nm to 100 nm from around 500 nm to 550 nm, and for triangles (ranging from 60 nm to 120 nm) from around 530 nm to 700 nm.

BET surface analysis revealed the non-porous character of TeaNPs (Fig. S2†). Recorded surface areas were 0.3272 ± 0.0230 m^2^ g^−1^ (*C* = 6.3) for B-TeaNPs, 2.2495 ± 0.1411 m^2^ g^−1^ (*C* = 7.2) for G-TeaNPs, and 0.2911 ± 0.0006 m^2^ g^−1^ (*C* = 5.2) for R-TeaNPs. Although G-TeaNPs were not the smallest (*cf.* SEM and DLS), they had the largest surface area due to irregular shapes.

HRTEM imaging revealed the presence of an organic layer covering the AgNPs. Its thickness on the surface of different TeaNPs was similar – around 2.5 nm (Fig. S3†). We used UV-vis, FTIR, and XPS spectroscopy to get information about the organic layer stabilizing TeaNPs.

The most interesting region of the UV-vis spectra corresponding to the polyphenolic species absorption is located between 240 and 310 nm. Among various polyphenolic compounds, isoflavonoids (especially catechins) seem the most important because their concentration in black and green tea extracts may be up to 5% or 10% of dry mass, respectively. Some catechins, namely epigallocatechin (EGC) and epigallocatechin gallate (EGCG), were proven to have antibacterial activity.^64^ According to the literature, the maxima of absorption for the most pronounced tea catechins are 273.6 nm for EGCG, 276.8 nm for ECG, 269.6 nm for EGC, and 278.4 for EC. Our tea extracts showed maximum absorption at 270 nm for black, 271 nm for red, and 273 nm for green tea extract (Fig. 1G), suggesting different content of catechins in all teas.^61^ Also, all spectra showed a very weak peak at around 350 nm which suggested slight oxidation of catechins.^62^ The situation changed in the case of TeaNPs prepared with adequate extracts (Fig. 1H). Both peaks located at around 270 nm (catechins) and 350 nm (oxidized catechins) were clearly visible, and their intensity ratio shifted towards oxidized moieties in TeaNPs.

FTIR analysis was conducted to identify the tea extracts' functional groups and confirm their presence in the TeaNPs (Fig. 2). All tea extracts had similar FTIR spectra. This was unsurprising because all the extracts contain polysaccharides, polyphenols, and caffeine (theine) as the main constituents.^63^ The differences in the spectra corresponding to green tea extract (Fig. 2B, additional peaks assigned to catechins, especially EGCG) were observed. These differences were also reflected in the corresponding FTIR spectra of TeaNPs. This, in turn, suggested a higher concentration of polyphenols on the surface of G-TeaNPs, which might result in better antimicrobial efficacy. A detailed discussion of FTIR results is presented in the ESI.†

The XPS analysis allowed us to estimate the chemical surface composition of TeaNPs. At the surface of all TeaNPs, silver, carbon, and oxygen were present, and their atomic percentages differed between TeaNP samples. Regardless of the sample, high atomic percentages of carbon and oxygen were detected (Fig. S2A†), suggesting large amounts of the organic compounds at the surface of TeaNPs. The 364.8 eV signal in high-resolution spectra of TeaNPs confirmed the presence of metallic silver in the samples (Fig. S2B†). Detailed information on surface composition is shown in Table S5.†

The XRD of TeaNPs (Fig. 1J–L) revealed peaks at a 2*θ* of 38.1°, 44.3°, 64.4°, and 77.4°, indicating the face-centered cubic crystalline structure of metallic silver (JCPDS file no. 01-071-4613). In cases of B-TeaNPs and R-TeaNPs, we observed an additional peak at 28°, suggesting the presence of silver oxide in the sample.

XRD showed the presence of silver oxide in B-TeaNPs and R-TeaNPs and not in G-TeaNPs. XPS indicated that on the surface of G-TeaNPs the amount of organic matter was larger when compared to B-TeaNPs and R-TeaNPs (Fig. S2A and B†). However, the imaging of TeaNPs with HRTEM suggested that the thickness of the organic layer was similar among the nanoparticles (Fig. S3†). Therefore, the amounts of polyphenolic compounds present in different tea extracts seemed to be more important than the thickness of the organic layer on the surface of TeaNPs. Seemingly different amounts of organic matter and no detection of Ag_2_O in XPS measurement can be explained by the fact that the chemical shift of silver is very similar for different states of this element. This means it was difficult to distinguish if the silver in a non-metallic state, detected by XPS, is Ag_2_O (detected by XRD) or the remains of AgNO_3_ from the synthesis. Moreover, the higher amount of catechins having antioxidant properties (EGC and EGCG) that are more abundant in green tea (as evidenced in FTIR analysis) might protect G-TeaNPs from oxidation.

A detailed description of XPS, XRD, BET, and HRTEM results is available in the ESI (Fig. S2 and S3†).

### Antibacterial properties

First, silver nanoparticles synthesized using tea extracts (B-TeaNPs, G-TeaNPs, and R-TeaNPs) were tested against representative strains of Gram-negative (*E. coli*) and Gram-positive bacteria (*E. faecium*). Gram-negative and Gram-positive bacteria have different nanoparticle susceptibilities due to differences in the cell envelope morphology (Fig. 3A).^65^ The control experiments were performed with citrate-capped AgNPs without tea extracts as a control (C-AgNPs). 1 mg mL^−1^, 0.5 mg mL^−1^, and 0.1 mg mL^−1^ were tested. The microbes were exposed to the nanoparticles at room temperature for three hours upon stirring, and the differences in the number of CFU per mL were recorded using the plating method. The results are presented in Fig. 3B. All TeaNPs were more effective than C-AgNPs. We chose 0.1 mg mL^−1^ for further testing, as at this concentration, the antibacterial activity did not result in 100% reduction, therefore allowing us to compare the action of different TeaNPs.

The synthesized TeaNPs were tested against representative strains of ESKAPE bacteria – *E. faecium*, *S. aureus*, *K. pneumoniae*, *A. baumannii*, *P. aeruginosa*, and *E. cloacae* (Fig. 3B), following the same protocol (three hours, room temperature, mixing, and 0.1 mg mL^−1^ of TeaNPs). The most pronounced decreases were observed for G-TeaNPs, ranging from around a 25% decrease in the number of bacterial cells of *E. faecium* to approximately a 90% decrease in the case of *E. cloacae*.

### Antibiotic and tea extract assay

We aimed to verify how TeaNPs compare to standard antibiotics. To evaluate this, *S. aureus* and *E. cloacae* were incubated for three hours with ampicillin at the minimum inhibitory concentration (0.01 mg mL^−1^) and 10× MIC (0.1 mg mL^−1^, which corresponded to the concentration of TeaNPs used in this study). The criterium for choosing these bacterial strains was their susceptibility to ampicillin. The studied *E. faecium*, *K. pneumoniae*, *A. baumannii*, and *P. aeruginosa* were multidrug-resistant strains. In a similar experiment, we aimed to enumerate the antibacterial effect of only tea extracts. This was done to show that the antibacterial effect of TeaNPs was due to the synergistic effect of both the metallic core and natural capping layer.

**Fig. 4:**
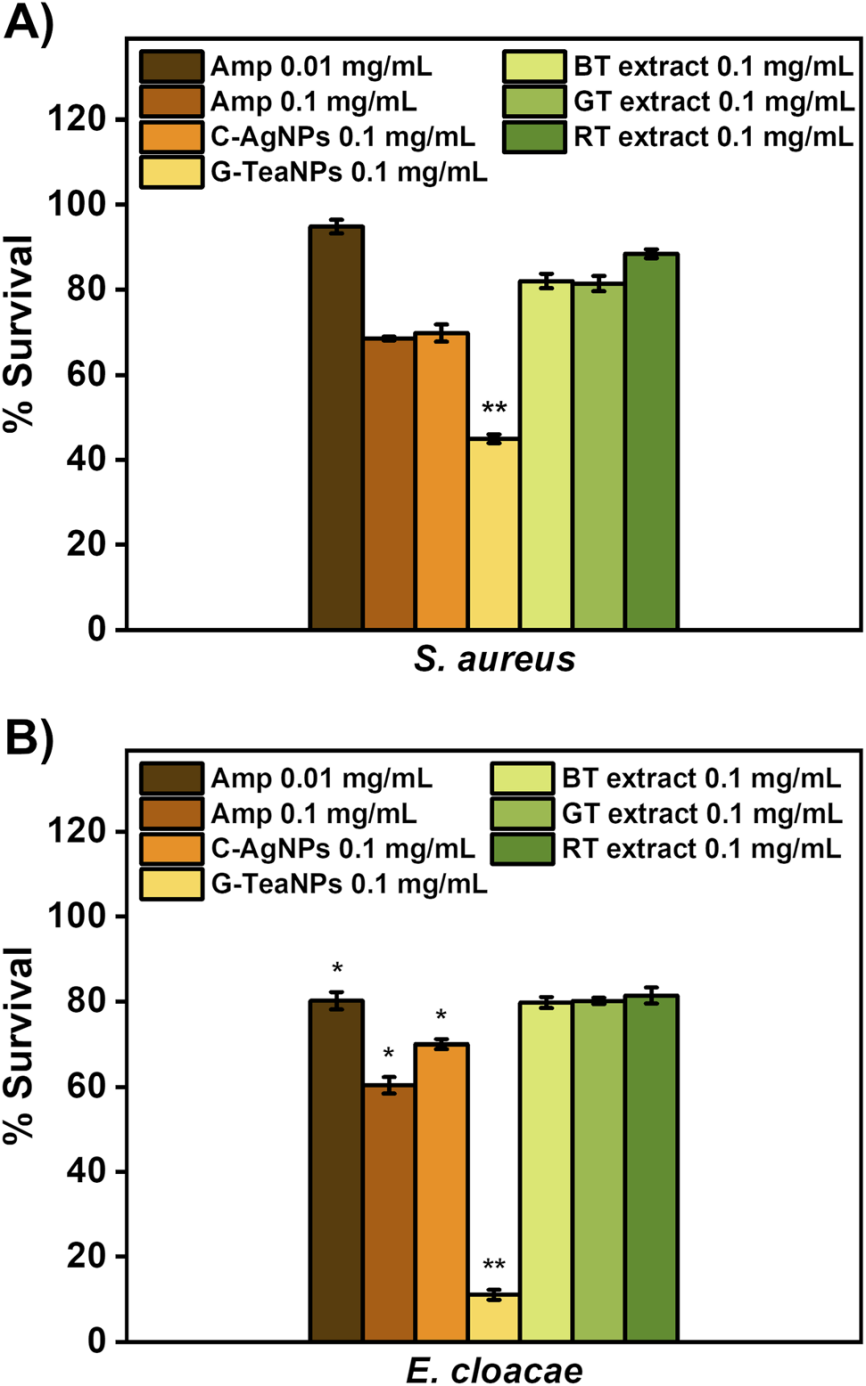
The comparison of antibacterial activity of ampicillin (MIC and 10× MIC), C-AgNPs, G-TeaNPs, and tea extracts at a concentration of 0.1 mg mL^−1^ against (A) *S. aureus* and (B) *E. cloacae*. The results are presented as a percentage of survival (*i.e.*, 
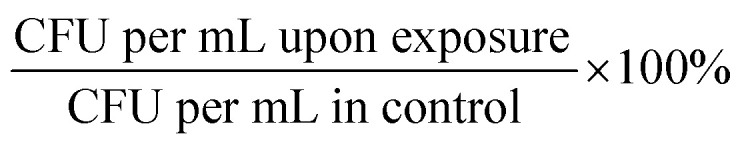
), *p < 0.05; **p < 0.01; ***p < 0.001, *p* values were calculated with respect to the control sample (not exposed to any AgNPs).

Tea extracts (black, green, and red) were dried using a rotary evaporator and suspended in 0.9% NaCl solution to reach a concentration of 0.1 mg mL^−1^. Then, *S. aureus* and *E. cloacae* were incubated for three hours with the tea extract solutions. The results are presented in Fig. 4. Tea extracts had a negligible effect on bacteria (not statistically significant). G-TeaNPs were more active than antibiotics against both tested bacterial strains at the same concentration (0.1 mg mL^−1^) and the same time of exposure (3 hours).

We also mixed C-AgNPs with tea extracts. Such mixtures showed lower antibacterial efficacy compared to the corresponding TeaNPs (Fig. S5†).

### Antifungal properties

To confirm the antifungal properties of TeaNPs, the multiresistant strains of *C. auris* and *C. neoformans* were used for the experiments. The tests were also performed with citrate-capped AgNPs without tea extracts as a control (C-AgNPs). To establish the minimal working concentration of the NPs, the experiments were performed at three different concentrations: 1 mg mL^−1^, 0.5 mg mL^−1^, and 0.1 mg mL^−1^. Yeast suspensions were exposed to the nanoparticles at room temperature for three hours, and the differences in the concentration of cells per mL were recorded using the plating method. The results are presented in Fig. 6.

All AgNPs were active against yeast, but G-TeaNPs were the most effective, causing around an 80% decrease in the number of living cells of *C. auris* at 0.5 mg mL^−1^ and around 90% decrease in the case of *C. neoformans* at 0.1 mg mL^−1^.

## Discussion

Silver nanoparticles are known for their antibacterial properties; to follow the eco-friendly trends of modern science, there's a need for synthesis protocols based on natural reducing agents such as plant extracts. Due to their antibacterial activity, tea extracts appear to be one of the most promising solutions for the green synthesis of nanoparticles. Different reports concerning silver nanoparticle synthesis using tea extracts are summarized in Table 1.

**Table 1:** The summary of the reports on using tea extracts for green synthesis of silver nanoparticles

Natural extract	Shape of NPs	Size (nm)	Application	Ref.
Green tea	Spherical	25 nm	Antibacterial	59
Red tea	Spherical	4 nm	—	68
Green tea	Spherical	5–30 nm	Sensing	69
Green tea	Spherical	44 nm	Antibacterial	53
Green tea	Spherical	20–90 nm	Antibacterial	70
Red tea	Spherical	11–30 nm	Antibacterial	52
Black tea and green tea	Spherical	20–25 nm	Antibacterial	71
Green tea	Spherical	15–20 nm	—	54
Green tea and garlic	Spherical	8 nm	Anticancer	72
Tea, unspecified	—	—	Antibacterial	73
Green tea	—	15–33 nm	Antibacterial	52
Green tea	Spherical	30 nm	Catalysis	74
Black tea and green tea	Spherical	21 nm	Antibacterial	75
Black tea, green tea, and red tea	Spherical and triangular	30–60 nm	Antibacterial and antifungal	Present work

Our study explores the possibility of improving antimicrobial properties using AgNPs capped with tea extracts (TeaNPs). We hypothesize that using tea extracts, known to have antimicrobial activity,^66^ allows for a synergistic effect when combined with AgNPs. In our study, as a control to which TeaNPs were compared, we used citrate-capped AgNPs. Citrates themselves have no significant effect on bacteria or yeasts.^67^ Green tea extract was selected due to its known antibacterial properties and widespread usage in eco-friendly AgNP synthesis. Black tea and red tea extracts were subjectively selected for comparison of the antimicrobial efficacy of different TeaNPs.

This study is the first attempt to verify the activity of tea-based AgNPs against a broad spectrum of pathogenic bacteria (complete ESKAPE panel) and yeasts. Multidrug-resistant strains of *E. faecium* (70/90; resistant to vancomycin, beta-lactams, and colistin, Table S1†), *A. baumannii* 2208, 81*, K. pneumoniae* 7K6, MCV37, and *P. aeruginosa* PA7 (sensitive only to carbapenems, Tables S2–S4†), were tested. *S. aureus*, *E. cloacae*, and *E. coli* strains were not drug-resistant. The studied strain of *C. auris* was resistant to amphotericin B.

At a concentration of 0.1 mg mL^−1^, all TeaNPs performed much better than C-AgNPs. For example, G-TeaNPs eradicated around 80% of *E. coli* (Fig. 3A), whereas C-AgNPs allowed only around 20% reduction. G-TeaNPs appeared to be the most effective against all examined bacteria, decreasing the bacterial titer by 60% to 90% for *S. aureus*, *K. pneumoniae*, *A. baumannii*, *P. aeruginosa*, *E. cloacae*, and *E. cloacae*. Only *E. faecium* (Fig. 3B) was less susceptible, with 16%, 23%, 12%, and 18% decreases upon exposure to G-TeaNPs, B-TeaNPs, R-TeaNPs, and C-AgNPs, respectively.

Tea extracts alone (not TeaNPs) at a concentration of 0.1 mg mL^−1^ didn't show significant antibacterial activity against *S. aureus* and *E. cloacae* (Fig. 4A and B). Such a result proved that natural extracts have insignificant antibacterial activity, as reported before.^76^

We also showed that the activity of TeaNPs, especially G-TeaNPs, was more pronounced than that of standard antibiotics. *S. aureus* and *E. cloacae* (non-resistant strains) were exposed to ampicillin at concentrations equal to the minimal inhibitory concentration (MIC; 0.01 mg mL^−1^) and 10× MIC (0.1 mg mL^−1^, *i.e.*, the same as the concentration of TeaNPs). For *S. aureus*, ampicillin at a concentration of 0.1 mg mL^−1^ presented antibacterial efficacy similar to that of C-AgNPs and R-TeaNPs (about 30% decrease in CFU per mL), but lower than that of G-TeaNPs and B-TeaNPs (around 60% decrease) (Fig. 3A). For *E. cloacae*, 0.1 mg mL^−1^ of ampicillin resulted in about 40% elimination of bacterial cells, slightly higher than the effect of C-AgNPs and R-TeaNPs. Again, for B-TeaNPs and G-TeaNPs, the observed elimination rates were higher, *i.e.*, 60% and about 90%, respectively (Fig. 3B). Adding black and green tea extracts enhanced their antibacterial effect, making nanoparticles even more efficient than certain antibiotics. Moreover, for *S. aureus*, we observed the antibacterial efficacy of C-AgNPs was comparable to the effectiveness of ampicillin in equal concentrations (0.1 mg mL^−1^) – bacterial survival was about 60%. For *E. cloacae*, this effect was less impressive, with about 70% survival, lower than that caused by 0.01 mg mL^−1^ ampicillin, but higher when compared to the efficacy of 0.1 mg mL^−1^ ampicillin. These observations confirmed that silver nanoparticles can be successfully used as an antibiotic alternative against Gram-negative and Gram-positive bacteria.

We expected to observe differences in the antimicrobial properties of TeaNPs against Gram-positive and Gram-negative bacteria due to the differences in the structure of their cell envelopes. The cell wall of Gram-positive bacteria is composed of a thicker layer of peptidoglycan compared to that of Gram-negative bacteria (about 20–80 nm *vs.* 1.5–10 nm).^65^ The thickness of the cell envelope makes Gram-positive bacteria generally less susceptible to nanoparticles.^77^ Alternatively, lipopolysaccharides on Gram-negative bacteria scavenge ions, small molecules, and toxins, thereby protecting cells.^78^

In our study, we observed a more significant antimicrobial effect of TeaNPs against the representatives of Gram-negative bacteria – *e.g.*, 80% elimination of *E. coli* compared to 20% elimination of *E. faecium* by using G-TeaNPs (0.1 mg mL^−1^) (Fig. 3A). This suggested that the main action of TeaNPs did not rely on the release of silver ions, while the presence of tea extracts contributed to their antimicrobial potential. The release of silver ions is only one possible mechanism of the antimicrobial properties of AgNPs. It is believed that AgNPs might also directly penetrate bacterial cell envelopes interacting with biomolecules such as proteins, nucleic acids, and lipids.^79^ The differences in the biocidal activity of TeaNPs against Gram-positive and Gram-negative bacteria strongly suggested that such a mechanism might occur in our case. The presence of large amounts of organic matter (mostly polyphenols) on the surface of TeaNPs most probably results in the additional delivery of polyphenolic compounds into the microbial cells. Therefore, the action of TeaNPs could be described as a ‘poisoned arrow’ mechanism”.

We used 10× MIC to compare the same concentration of both agents (antibiotics and TeaNPs) after the same incubation times (three hours). In some tests, *e.g.*, to find the minimum duration for killing 99% of the population (MDK_99_),^80^ the concentration of the applied antibiotic is usually at least 20× MIC, reaching even 100× MIC.^81^ In the case of ampicillin, the MDK_99_, after around ∼3 hours, used a concentration of at least 0.2 mg mL^−1^ (MIC = 0.01 mg mL^−1^).

In most protocols employing silver nanoparticles, only minimal inhibitory concentration (MIC) is estimated,^53,82–85^ similar to antibiotic antibacterial assays. However, MIC-based metrics are limited to qualitative analysis of a particular substance.^86^ Also, such protocols always provide information on microbial survival after *de facto* overnight (about 16 hours) exposure to the active substance.

**Fig. 5:**
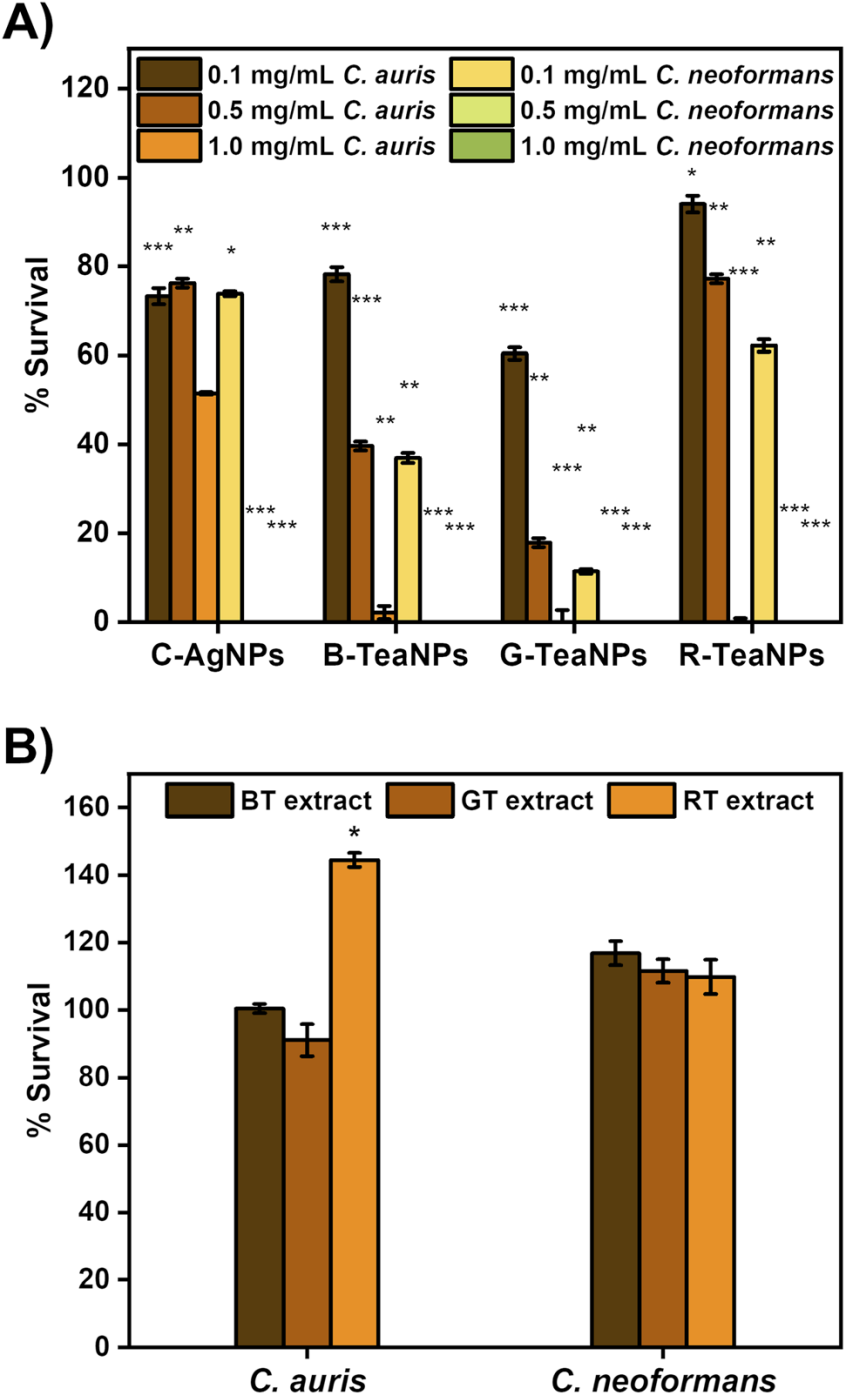
(A) Dose compensation of TeaNPs against *C. auris* and *C. neoformans* with three concentrations: 0.1 mg mL^−1^, 0.5 mg mL^−1^, and 1 mg mL^−1^. (B) Antimicrobial activity of tea extracts (0.5 mg mL^−1^) against *C. auris* and *C. neoformans*. The results are presented as a percentage of survival (*i.e.*, 
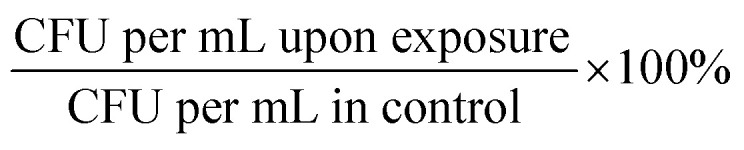
), *p < 0.05; **p < 0.01; ***p < 0.001, *p* values were calculated with respect to the control sample (not exposed to any AgNPs).

Next, we investigated the antifungal properties of TeaNPs. The structure of yeast cells differs from that of bacterial cells. The cell envelope of yeast is composed of beta-glucans, chitin, and mannoproteins, which are believed to be unaffected by silver.^77^ Therefore, antibacterial properties do not imply antifungal activity. The number of reports on successfully using silver nanoparticles against yeast is limited.^87–89^ We observed an antifungal effect of TeaNPs against yeasts, represented by *C. neoformans*, and the emerging opportunistic pathogen *C. auris* (in this study, we used a strain resistant to amphotericin B and azole drugs). Both species are clinically significant due to the severe infection trait (*C. neoformans*)^90^ or rapidly developing drug resistance (*C. auris*).^34^ At 1 mg mL^−1^ concentration, all examined AgNPs (including C-AgNPs) presented an elimination rate close to 99%. However, at lower concentrations of 0.1 mg mL^−1^ and 0.5 mg mL^−1^, C-AgNPs allowed only around a 25% decrease. At a concentration of 0.5 mg mL^−1^, B-TeaNPs and G-TeaNPs reduced the number of *C. auris* cells by about 60% and 80%, respectively. 0.1 mg mL^−1^ of TeaNPs was sufficient to cause a significant decrease in the number of *C. neoformans* but not *C. auris* cells. After 3 hours of exposition, G-TeaNPs resulted in about 80% elimination of *C. neoformans*; for B-TeaNPs, the elimination rate was about 60%. C-AgNPs and R-TeaNPs allowed for about 20–30% of titer reduction. At the concentrations of 0.5 mg mL^−1^ and 1.0 mg mL^−1^, the number of remaining cells was below the detection limit (∼10 cells per mL) for all studied AgNPs (including C-AgNPs) (Fig. 5A). We also performed a control experiment, where we did not observe any significant antifungal activity of tea extracts against yeast cells (Fig. 5B). In the case of *C. auris*, a value above 100% against RT extract was most likely used by cells metabolizing the components of the extracts. In fact, tea extracts alone were found to not only be ineffective against *Candida* spp.,^91^ but also vital for fungal biofilm formation.^92,93^

**Fig. 6:**
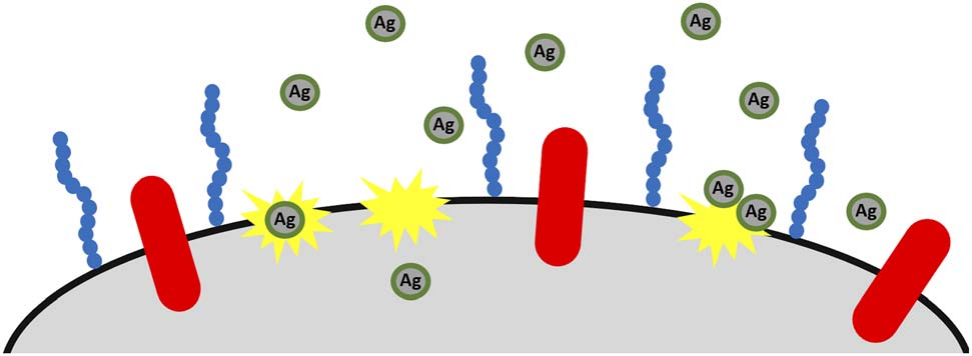
Schematic illustration of antibacterial activity of TeaNPs. AgNPs capped with tea extract components compromise the integrity of bacterial envelopes and enter the cell, causing further damage to the cell's functioning (‘poisoned arrow’ mechanism).

These findings suggest that utilizing tea extracts allows for applying AgNPs against yeasts;^77,94^ at lower concentrations, *i.e.*, 0.5 mg mL^−1^ or even 0.1 mg mL^−1^ in some cases. *C. auris* required higher concentrations than *C. neoformans*. It is possible that the exposure to TeaNPs caused *C. auris* to enter an arrested state, thereby promoting the development of a resistance mechanism similar to that developed after exposure to fungicidal drugs. This is frequently the cause for higher MDK values for fungicidal drugs compared to antibiotics.^95^ Nevertheless, TeaNPs can be successfully used for the prevention of fungal infections. However, due to the differences in antimicrobial efficacy against different groups of fungi, a concentration of 0.5 mg mL^−1^ is suggested.

AgNPs are believed to act *via* different phenomena, including the generation of ROS, disruption of the replication of DNA, increasing cell membrane permeability, penetration of cell envelopes, alteration of the cell membrane structure, and agglomeration on the cell's surface, disrupting the integrity of cell envelopes or cell division.^79^ Disrupting the integrity of cell envelopes may adequately explain the antifungal activity of TeaNPs against the representatives of yeast and why the efficient elimination of fungi requires a higher concentration of AgNPs.

The differences between particular AgNPs most probably depend on the concentration of polyphenols and isoflavonoids, just as in the case of bacteria. The antibacterial action of TeaNPs is presented schematically in Fig. 6.

Other researchers showed that the shape of nanoparticles has an impact on cytotoxicity.^46,96,97^ In our study, we observed spontaneously formed triangle-shaped B-TeaNPs and irregular (also triangular) G-TeaNPs. The greater variety of shapes than in C-AgNPs and R-TeaNPs (primarily spherical) might also result in more significant antimicrobial activity. Undoubtedly, different compositions of various tea extracts affected this parameter.

The size of nanoparticles is usually related to the cytotoxic effect of nanomaterials,^98^ with smaller particles being more cytotoxic. This should favor C-AgNPs and R-TeaNPs over G-TeaNPs and B-TeaNPs in our experiments. This was not the case. In most experiments, C-AgNPs and R-TeaNPs showed the lowest antimicrobial efficacy. This is in line with other studies, which demonstrated that size is not a primary factor affecting the antimicrobial activity of AgNPs.^97,99–102^

XRD allowed us to confirm not only the presence of face-centered cubic crystalline planes of metallic silver in all TeaNP samples but also the presence of silver oxide (Ag_2_O) in B-TeaNPs and R-TeaNPs (Fig. 1J–L). The presence of silver oxide on the surface of B-TeaNPs and R-TeaNPs may explain their weaker antimicrobial activity when compared to G-TeaNPs. For G-TeaNPs, no or insignificant traces of silver oxide were found. The XPS analysis of the chemical surface composition of TeaNPs indicated a large amount of organic compounds on the surface of TeaNPs, along with the presence of metallic silver (Fig. S2A and B†). Moreover, the increased surface area of G-TeaNPs, estimated with BET theory, may be another factor responsible for their increased antimicrobial properties compared to those of B-TeaNPs and R-TeaNPs. All these findings suggest the successful synthesis of silver nanoparticles using tea extracts and a large amount of organic matter on the surface of TeaNPs, which was confirmed with HRTEM imaging (Fig. S3†). Hence the thickness of the organic layer on the surface of different TeaNPs is similar, and the composition of organic compounds in the tea extracts seems to be the determining factor for the improvement of antimicrobial activity. Among various polyphenolic compounds, isoflavonoids (especially catechins) seem the most important. Higher concentrations of polyphenols, along with EGC and EGCG, may be a reason for the good performance of B-TeaNPs and G-TeaNPs in eliminating bacteria.

Finally, we found that the antimicrobial potential of synthesized TeaNPs depends strongly on the selected method for synthesis. We repeated some antibacterial tests for two other sets of nanoparticles synthesized using other previously reported synthesis protocols.^68,103^ The data are available in the ESI.† Among the three selected synthesis methods, only one allowed for synthesizing efficient antimicrobial TeaNPs (Fig. S4†). Moreover, the enhanced antimicrobial activity of TeaNPs was more than the additive effect of tea extracts and AgNPs. Hence TeaNPs presented higher antibacterial efficacy when compared to the mixtures of C-AgNPs and tea extracts (Fig. S5†).

The cytotoxicity of AgNPs is a significant drawback when it comes to their application for therapeutic purposes. In the study by Arumai Selvan *et al.*, the authors compared the cytotoxicity of AgNPs synthesized using garlic and tea extracts against five cell lines: NHDF, MCF-7, HeLa, Hep-2, and A549. Depending on the set of nanoparticles, green tea-AgNPs caused about 20% growth inhibition of NHDF cells (normal human dermal fibroblasts) and 70–80% growth inhibition of cancer cell lines (MCF-7, HeLa, Hep-2, and A549). The cytotoxicity of green tea AgNPs was also estimated as IC_50_ values (concentration required for 50% growth inhibition) for each cell line. For the cancer cell lines, the IC_50_ values were about 20 μg mL^−1^, and for NHDF cells, this value was >100 μg mL^−1^. For chemically synthesized AgNPs, IC_50_ values were 22–32 μg mL^−1^, but the cell growth inhibition among all tested cell lines was comparable to the effect of tea-AgNPs.^72^ Therefore the cytotoxicity of AgNPs synthesized using tea extracts is relatively high yet comparable to that of common chemically synthesized AgNPs. Moreover, the study by Rolim *et al.* reported the differences in the viability of HaCat cells by about 20% after exposure to green tea AgNPs at concentrations up to 30 μg mL^−1^.^53^

## Conclusions

We established that silver nanoparticles synthesized with tea extracts have higher antibacterial properties than silver nanoparticles alone. Therefore, lower dosages of TeaNPs could be used (0.1 mg mL^−1^). We confirmed that the synergistic effect of tea extracts and silver nanoparticles allowed for efficacy higher than that of antibiotics (ampicillin) when tested at the same concentrations (0.1 mg mL^−1^) and after a relatively short exposure time of three hours. The efficacy of TeaNPs was proved against emerging bacterial (ESKAPE group) and fungal pathogens (*C. neoformans* and *C. auris*). No previously published reports examined the antimicrobial properties of AgNPs on such a wide range of microorganisms. Moreover, we showed how important a proper protocol is for NP synthesis, for the antimicrobial activity of TeaNPs depends on it. However, the matter of cytotoxicity of AgNPs remains a drawback to be overcome in future studies.

## Author contributions

S. R. & M. W.: conceptualization, writing the first draft, original draft preparation, conducting experiments, analysis of data, editing; M. G.: conducting experiments, writing the first draft, editing; P. M.: original draft preparation, editing, discussion of data; W. A.: conducting experiments, discussion of data, original draft preparation; K. N.: conducting experiments, discussion of data; J. P.: conceptualization, supervision, investigation, discussion of data, writing—review, and project administration.

## Conflicts of interest

The authors declare no conflict of interest.

## Supplementary Material

NA-005-D3NA00220A-s001

NA-005-D3NA00220A-s002
