# Supplementary material for: Enhancing the antimicrobial activity of silver nanoparticles against ESKAPE bacteria and emerging fungal pathogens by using tea extracts
Source: Nanoscale Adv. 2023 Aug 23;5(21):5786–98. doi: 10.1039/d3na00220a (PMC10597549; doi:10.1039/d3na00220a)
Supplement: NA-005-D3NA00220A-s002 [file NA-005-D3NA00220A-s002.pdf]

Electronic Supplementary Material (ESI) for Nanoscale Advances.
This journal is © The Royal Society of Chemistry 2023

Enhancing the antimicrobial activity of silver nanoparticles against ESKAPE bacteria and emerging fungal pathogens by using tea extracts

Sada Raza[1], Mateusz Wdowiak[1], Mateusz Grotek[1,2], Witold Adamkiewicz[1], Kostiantyn Nikiforow[1], Pumza Mente[1], Jan Paczesny[1,*]

Table of content

1. Chemicals used in the study……1
2. Green synthesis protocols of TeaNPs……1
3. Drug-resistance profiles of bacterial strains……2
4. Characterization of citrate capped AgNPs……7
5. FTIR analysis of tea extracts and TeaNPs……7
6. XRD, XPS, BET, and HRTEM analysis of TeaNPs…… 7
7. A comparison of antimicrobial activity obtained by using different TeaNPs synthesis methods……8

1. *Chemicals used in the study*

Liquid LB medium contained 10 g/L NaCl, 10 g/L tryptone, and 5 g/L yeast extract. Liquid YPD medium contained 20 g/L casein extract, 10 g/L yeast extract, and 20 g/L glucose. LB-agar and YPD-agar were LB and YPD supplemented with 15 g/L of agar, respectively. All the media were purchased as pre-mix (Carl Roth, Germany). For bacteria and yeasts suspensions dilutions, a 0.9% solution of NaCl (Sigma-Aldrich, > 99%, estimated by $AgNO_3$ titration) in Milli-Q water was used.

For the synthesis of AgNPs, silver nitrate (ACS reagent > 99.0%, estimated by KSCN titration), sodium borohydride (powder, >98%), sodium hydroxide (reagent grade, >98%, estimated by HCl titration), and trisodium citrate, TSC (>98%, estimated by GC analysis) were purchased from Sigma Aldrich and used as received without further purification. Milli-Q Water (Merck; 18.2 MΩ·cm) was used to prepare all solutions and extracts.

2. *Green synthesis protocols of TeaNPs*

TeaNPs were synthesized by three different protocols but with the same raw materials. The first protocol [1] is described in the main manuscript, while descriptions of the other methods are as follows:

Synthesis 2 [2]: silver nanoparticles with tea extracts were synthesized using $AgNO_3$ and tea extracts. In this method, three kinds of tea: black (Loyd, Mokate, Poland), green (Loyd, Mokate, Poland), and Pu-erh (Lord Nelson, Lidl, Poland), were frozen in liquid nitrogen and ground using a mortar and pestle. The tea extracts were prepared by weighing 10 g of ground tea leaves in a 500 mL beaker along with 100 mL of distilled water and maintained at 60°C for 10 min before decanting it. The solution was filtered by a 0.45 µm Millipore membrane filter followed by a 0.2 µm Millipore membrane filter. To synthesize silver nanoparticles, 100 mL of $AgNO_3$ (1 mM) was reacted with 12 mL of the tea extract in the Erlenmeyer flask at room temperature. The color changes of the solution were observed, indicating the formation of nanoparticles.

Synthesis 3 [3]: silver nanoparticles with tea extracts were synthesized using $AgNO_3$ and tea extracts. In this method, three kinds of tea: black (Loyd, Mokate, Poland), green (Loyd, Mokate, Poland), and Pu-erh (a type of red tea; Lord Nelson, Lidl, Poland), were frozen in liquid nitrogen and ground using a mortar and pestle. 400 mg of the ground leaves were added to 30 mL of water. The mixture was vigorously stirred over a magnetic stir plate for 30 min at room temperature and filtered with Whatman 1 filter paper. The stock solution of the tea leaf extract was diluted to 5%, 10%, 20%, 30%, and 40% (V/V) with deionized water. Next, 1 mL of silver nitrate (1 mM) and 0.25 mL of NaOH (0.10 M) solutions were added dropwise in sequence into the dilute tea extract solutions. The contents of the reaction vessels were combined with gentle swirling, and brown AgNPs were produced immediately.

3. *Drug-resistance profiles of bacterial strains*

Table S1. Antibiotic resistance of *Enterococcus faecium* DSM 13590 strain, presented as a diameter of the inhibition zone in the disc test. Complete resistance was marked with red color, intermediate resistance with light red color, and susceptibility with white color. The table was prepared based on the data from the Bacdive database (https://bacdive.dsmz.de/strain/5299).

	Test 1	Test 2	Test 3
	Inhibition zone (mm)	Inhibition zone (mm)	Inhibition zone (mm)
Penicillin G	0	0	0
Oxacillin	0	0	0
Ampicilin	12	12	12
Ticarcillin	0	0	0
Mezlocillin	10	10	10
Cefatolin	0	0	0
Cefatozin	0	0	0
Cefotaxime	0	0	0
Aztreonam	0	0	0
Imipenem	6	10	10
Tetracycline	10	0	0
Chloramphenicol	24	22	22
Gentamycin	12	12	12
Amikacin	12	14	14
Vancomycin	0	0	0
Erythromycin	0	0	0
Lincomycin	0	0	0
Ofloxacin	18	18	18
Norfloxacin	20	20	20
Colistin	0	0	0
Pipemidic acid	0	0	0
Nitrofuratoin	12	10	10
Bacitracin	0	0	0
Polymyxin B	0	0	0
Kanamycin	10	12	12
Neomycin	14	14	14
Doxycycline	18	16	16
Ceftriaxone	0	0	0
Clindamycin	0	0	0
Fosfomycin	20	20	20
Moxifloxacin	26	24	24
Linezolid	30	28	28
Nystatin	0	0	0
Quinupristin/Dalfopristin	18	18	18
Teicoplanin	6	0	0
Piperacillin/Tazobactam	6	6	6

Table S2. Antibiotic resistance of *Acinetobacter baumannii* ATCC 19606 strain, presented as a diameter of the inhibition zone in the disc test. Complete resistance was marked with red color, intermediate resistance with light red color, and susceptibility with white color. The table was prepared based on the data from the Bacdive database (https://bacdive.dsmz.de/strain/8093).

	Test 1	Test 2
	Inhibition zone (mm)	Inhibition zone (mm)
Penicillin G	0	0
Oxacillin	0	0
Ampicillin	0	0
Ticarcillin	22	24
Mezlocillin	12	10
Cefatolin	0	0
Cefatozin	0	0
Cefotaxime	12	12
Aztreonam	10	10
Imipenem	24	36
Tetracycline	24	24
Chloramphenicol	6	0
Gentamycin	14	16
Amikacin	20	22
Vancomycin	0	0
Erythromycin	12	10
Lincomycin	0	0
Ofloxacin	28	26
Norfloxacin	18	16
Colistin	16	16
Pipemidic acid	0	0
Nitrofuratoin	6	6
Bacitracin	0	0
Polymyxin B	16	18
Kanamycin	24	24
Neomycin	18	20
Doxycycline	30	30
Ceftriaxone	14	14
Clindamycin	0	0
Fosfomycin	12	10
Moxifloxacin	28	28
Linezolid	0	0
Nystatin	0	0
Quinupristin/Dalfopristin	0	0
Teicoplanin	0	0
Piperacillin/Tazobactam	24	24

Table S3. Antibiotic resistance of *Klebsiella pneumoniae* ATCC 700603 strain, presented as a diameter of the inhibition zone in the disc test. Complete resistance was marked with red color, intermediate resistance with light red color, and susceptibility with white color. The table was prepared based on the data from the Bacdive database (https://bacdive.dsmz.de/strain/8093).

	Test 1	Test 2	Test 3
	Inhibition zone (mm)	Inhibition zone (mm)	Inhibition zone (mm)
Penicillin G	0	0	0
Oxacillin	0	0	0
Ampicillin	0	12	12
Ticarcillin	0	0	0
Mezlocillin	0	10	10
Cefatolin	0	0	0
Cefatozin	0	8	10
Cefotaxime	20	0	0
Aztreonam	0	10	10
Imipenem	36	34	36
Tetracycline	20	14	16
Chloramphenicol	26	12	8
Gentamycin	10	14	16
Amikacin	26	24	26
Vancomycin	0	0	0
Erythromycin	6	6	0
Lincomycin	0	0	0
Ofloxacin	18	18	18
Norfloxacin	20	20	20
Colistin	5	5	5
Pipemidic acid	16	12	14
Nitrofuratoin	12	10	10
Bacitracin	0	0	0
Polymyxin B	20	16	16
Kanamycin	0	14	14
Neomycin	10	14	16
Doxycycline	10	10	10
Ceftriaxone	16	20	20
Clindamycin	0	0	0
Fosfomycin	14	16	16
Moxifloxacin	18	20	20
Linezolid	0	0	0
Nystatin	0	0	0
Quinupristin/Dalfopristin	0	0	0
Teicoplanin	0	0	0
Piperacillin/Tazobactam	18	18	18

Table S4. Antibiotic resistance of *Pseudomonas aeruginosa* DSM 24068 strain, presented as a diameter of the inhibition zone in the disc test. Complete resistance was marked with red color, intermediate resistance with light red color, and susceptibility with white color. The table was prepared based on the data from the Bacdive database (https://bacdive.dsmz.de/strain/12794).

	Test 1	Test 2
	Inhibition zone (mm)	Inhibition zone (mm)
Penicillin G	0	0
Oxacillin	0	0
Ampicillin	0	0
Ticarcillin	6	6
Mezlocillin	0	0
Cefatolin	0	0
Cefatozin	0	0
Cefotaxime	0	0
Aztreonam	6	6
Imipenem	30	30
Tetracycline	16	16
Chloramphenicol	0	0
Gentamycin	0	0
Amikacin	18	18
Vancomycin	0	0
Erythromycin	0	0
Lincomycin	0	0
Ofloxacin	0	0
Norfloxacin	0	0
Colistin	16	16
Pipemidic acid	0	0
Nitrofuratoin	0	0
Bacitracin	0	0
Polymyxin B	18	18
Kanamycin	0	0
Neomycin	0	0
Doxycycline	12	12
Ceftriaxone	0	0
Clindamycin	0	0
Fosfomycin	0	0
Moxifloxacin	0	0
Linezolid	0	0
Nystatin	0	0
Quinupristin/Dalfopristin	0	0
Teicoplanin	0	0
Piperacillin/Tazobactam	0	0

4. *Characterization of citrate-capped AgNPs*

UV-Vis absorption spectra were recorded using a Thermo Scientific Evolution 220 spectrometer with a temperature controller. The measurements were carried out using 1 cm quartz cuvettes (Hellma, Germany).

The hydrodynamic sizes of the NPs were determined using the dynamic light scattering (DLS) technique. The DLS measurements were conducted with the Malvern ZetaSizer Nano-ZS instrument using 1 cm quartz cuvettes (Hellma, Germany).

Scanning electron microscope (SEM) images were collected with a Nova NanoSEM 450 under a high vacuum (10^{-7} mbar). The purified samples were deposited on a silicon substrate, allowed to dry, and mounted onto the standard SEM stub with carbon tape. The images were collected using the Through Lens Detector (TLD) of secondary electrons at a primary beam energy of 10 kV and a 4.8 mm working distance. The average diameters of the NPs were calculated from the SEM images using the ImageJ software by measuring at least 100 particles per sample.

Fourier-transformed infrared spectroscopy (FTIR) studies were performed with a Vertex 80 V FTIR spectrometer (Bruker, USA) equipped with a Platinum ATR (Bruker, USA) module. The tea extracts were dried by rotary evaporation at 65 °C to remove water. The TeaNPs were centrifuged and then dried in the oven at 65 °C. Dried powder samples were placed on a diamond prism (1 mm x 1 mm) to cover it entirely. The spectral resolution of the measurement was 2 cm^{-1}, and the number of scans was 64.

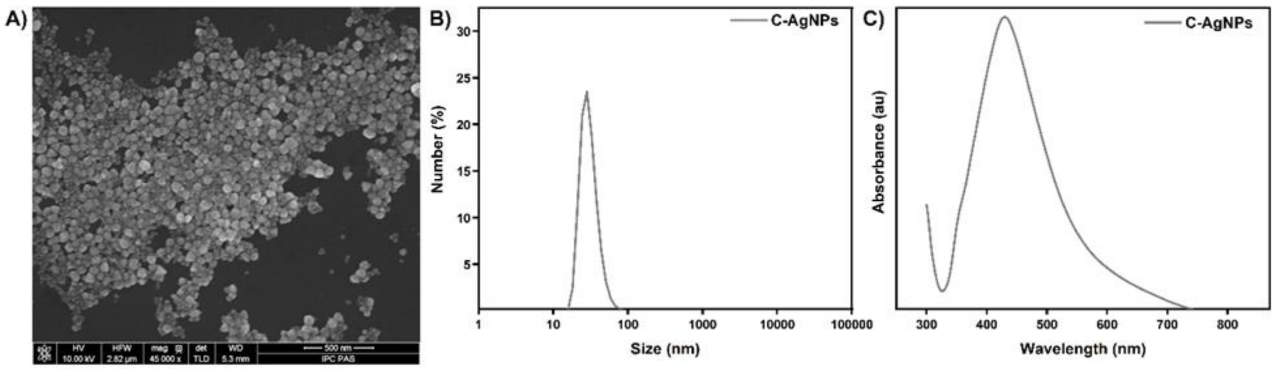

Figure S1. The characterization of control citrate capped AgNPs (C-AgNPs). **A)** SEM image of C-AgNPs; **B)** DLS size estimation of C-AgNPs; **C)** UV-Vis spectrum of C-AgNPs.

The characterization of C-AgNPs showed the sizes of synthesized nanoparticles estimated by means of SEM (**Figure S1A**), DLS (**Figure S1B**), and UV-Vis spectrum (**Figure S1C**). The diameters of C-AgNPs were approximately about 30 nm, so similar to the sizes of R-TeaNPs (34 ± 7 nm, **Figure 1**). In the UV-Vis absorption spectrum, the maximum absorption was observed for the wavelength of about 420 nm.

5. *FTIR analysis of tea extracts and TeaNPs*

To characterize the tea extracts and their interaction with silver nanoparticles (forming TeaNPs), Fourier-transform infrared (FTIR) spectroscopy was used. All tea extracts spectra shown in **Figure 2** are in accordance to literature data of flavanols [4].

The peak at 2925 cm^{-1} is often attributed to asymmetric stretching in aliphatic saturated groups, while the region around 1000 cm^{-1} corresponds to ring breathing modes. Most of the bands were assigned to carbonyl group vibration (1604 and 1519 cm^{-1}), 1446 cm^{-1} (phenyl ring), C-O stretching vibration of an aromatic alcohol (1144 cm−1), and C-O-C stretching vibration (1040 cm^{-1}). The region of 900 – 750 cm^{-1} is dominated by wagging vibration of hydroxyl groups. The differences among catechins are mostly connected with the position of OH group e.g. for (+)- catechin and gallocatechin, which structures differ in presence of hydroxyl group and spectra exhibit differences in bands related to OH and CO vibrations. Band characteristic for OH stretching forms broad peak from 1373 to 1280 cm^{-1} for gallocatechin. Gallocatechin IR spectrum also contain additional band at 740 cm^{-1} assigned to out of plane wagging of CH in aromatic ring. The most striking is the presence of band at 1698 cm^{-1} assigned to ester C=O in the case of EGCG. Other bands characteristic for aromatic C=O are bands at 1603 cm−1, 1558 cm−1, and 1450 cm−1. While, the bands around 1238 cm−1, 1144 cm−1, and 1014 cm−1 can be assigned to vibration of aromatic O-H, antisymmetric stretching of C-O-C, alcohol C–OH, and C-O, respectively. The FT-IR bands characteristic for epicatechin 1623 cm−1, 1520 cm−1, 1470 cm−1 – C=O (phenyl ring), 1142 cm−1 and 1041 cm−1 (C-O-C stretching vibration).

All spectra show expected mixture of peaks for different catechins with EGCG being the most prominent in green tea extract spectrum.

For each set of TeaNPs, significant differences in FTIR, compared to the spectra of adequate tea extracts, were observed with the G-TeaNPs being the most catechin rich.

6. *XRD, XPS, BET, and HRTEM analysis of TeaNPs*

For further understanding of TeaNPs formulation, X-ray diffraction (XRD) and X-ray photoelectron spectroscopy (XPS) were performed. Additionally, we examined the physical adsorption of gas molecules on TeaNPs according to Brunauer–Emmett–Teller (BET) theory.

X-ray diffraction analysis (XRD) was performed with an Empyrean range diffractometer (Malvern PANalytical, Malvern, United Kingdom). Dried powder samples were placed on the measurement table to cover it entirely. Measurement was performed at room temperature with the wavelength λ = 0.154 nm, using 1/4 and 1/8 diaphragms. The total time of measurement was 72 minutes. The XRD pattern of the

dried TeaNPs (**Figure 1J-L)** indicates XRD peaks at 2θ degree of 38.1, 44.3, 64.4, and 77.4, attributed to the (1 1 1), (2 0 0), (2 2 0), and (3 1 1) crystalline planes. This indicates the face-centered cubic crystalline structure of metallic silver (JCPDS file No. 01-071-4613) [5]. Moreover, in the diffractograms of B-TeaNPs and R-TeaNPs, the peaks near 2θ degree of 28 and 31.9, attributed to (1 1 0) and (1 1 2) crystalline plates adequately were present, suggesting the presence of Ag_2O in these samples [5]. However, these signals may also come from silver nitrate. The intensity of these peaks was much lesser in the G-TeaNPs diffractogram, so it's hard to distinguish it from the background signal. The differences in the content of Ag_2O, which is less reactive than metallic silver, between B-TeaNPs, G-TeaNPs, and R-TeaNPs is probably an explanation for the antimicrobial efficacy of TeaNPs – G-TeaNPs with the least amount of non-metallic silver appeared to be the best performing in the antimicrobial assay.

X-ray photoelectron spectroscopy (XPS) was performed with CLAM2 XPS Spectrometer (VG Microtech Ltd, London, United Kingdom). Dried powder samples were placed in the measurement table to cover the appropriate surface. Measurement was performed in a vacuum, within the binding energy ranging from 0 – 1300 eV. The elemental analysis allowed for the estimation of atomic percentages of B-TeaNPs, G-TeaNPs, and R-TeaNPs. In the samples, high amounts of carbon (39.18% – 72.71%) and oxygen (24.86% - 36.3%) were detected, while the amount of silver on the surface of the nanoparticles was significantly lower (0.85% - 16.13%). The analysis also indicated the presence of certain amounts of nitrogen (1.48% - 6.5%) and potassium (0.12% - 1.89%) (**Figure S2A**). In the high-resolution spectra of all examined TeaNPs samples, signals of about 368.4 eV were detected, confirming the presence of metallic silver in the nanoparticles samples (**Figure S2B**). The detailed information of atomic percentages on the surface of TeaNPs were placed in **Table S5**. All these findings suggest the successful synthesis of silver nanoparticles using tea extracts and the large amount of organic matter present on the surface of TeaNPs. A certain amount of silver nitrate may be present in the samples.

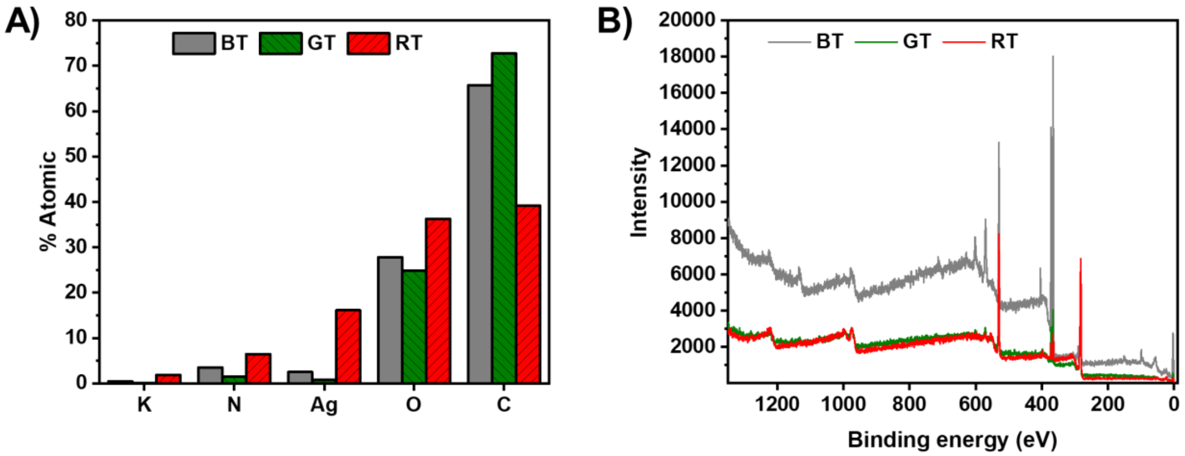

Figure S2. A) Elemental XPS analysis of B-TeaNPs, G-TeaNPs, and R-TeaNPs; B) High-resolution spectra of B-TeaNPs, G-TeaNPs, and R-TeaNPs.

Table S5. A detailed characterization of TeaNPs surface composition estimated using XPS spectroscopy. The results were presented as atomic percentages.

B-TeaNPs				G-TeaNPs				R-TeaNPs			
Name	Position	FWHM	%At Conc	Name	Position	FWHM	%At Conc	Name	Position	FWHM	%At Conc
**O 1s a**	531.58	1.86	**6.54**	**O 1s a**	531.44	1.76	**2.67**	**O 1s a**	530.92	1.64	**2.14**
**O 1s b**	532.94	1.86	**20.68**	**O 1s b**	532.91	1.76	**22.19**	**O 1s b**	532.61	1.64	**34.16**
**O 1s c**	535.12	1.86	**0.57**								
			**27.79**				**24.86**				**36.3**
**N 1s a**	400.21	2.54	**3.16**	**N 1s**	400.47	2.18	**1.48**				
**N 1s b**	406.64	1.5	**0.37**					**N 1s**	406.86	1.09	**6.5**
			**3.53**				**1.48**				**6.5**
**Ag 3d 5/2**	368.36	1.43	**1.52**	**Ag 3d 5/2**	368.44	1.37	**0.51**	**Ag 3d 5/2**	368.52	1.18	**9.68**
**Ag 3d 3/2**	374.36	1.43	**1.01**	**Ag 3d 3/2**	374.44	1.37	**0.34**	**Ag 3d 3/2**	374.52	1.18	**6.45**
			**2.53**				**0.85**				**16.13**
**C 1s a**	284.65	1.4	**24.87**	**C 1s a**	284.7	1.32	**35.41**	**C 1s a**	284.67	1.33	**20.93**
**C 1s b**	285.64	1.4	**11.29**	**C 1s b**	285.63	1.32	**7.49**	**C 1s b**	285.44	1.33	**5.31**
**C 1s c**	286.51	1.4	**19.03**	**C 1s c**	286.43	1.32	**22.67**	**C 1s c**	286.54	1.33	**7.25**
**C 1s d**	287.8	1.4	**6.57**	**C 1s d**	287.8	1.32	**4.49**	**C 1s d**	287.84	1.33	**3.59**
**C 1s e**	288.99	1.4	**3.95**	**C 1s e**	288.89	1.32	**2.65**	**C 1s e**	289.06	1.33	**2.1**
			**65.71**				**72.71**				**39.18**
**K 2p 3/2**	293.05	1.33	**0.28**	**K 2p 3/2**	293.39	0.88	**0.12**	**K 2p 3/2**	293	1.15	**1.26**
**K 2p 1/2**	296.07	1.33	**0.14**					**K 2p 1/2**	295.8	1.15	**0.63**
			**0.42**				**0.12**				**1.89**

The surface areas of tested materials were measured with an ASAP 2020 automatic analyzer (Micromeritics Instrument Corp., USA), using krypton as an adsorbate. Before adsorption measurements at liquid nitrogen temperature (77 K), the samples were outgassed at 373K for 15 hours in a vacuum chamber to clean their surface. The specific surface area of AgNPs were calculated using the BET (Brunauer–Emmett–Teller) method. The analysis revealed the surface areas of TeaNPs were as follows:

0.3272 +/- 0.0230 m^2/g (C = 6.3) for B-TeaNPs, 2.2495 +/- 0.1411 m^2/g (C = 7.2) for G-TeaNPs, and 0.2911 +/- 0.0006 m^2/g (C = 5.2) for R-TeaNPs, with the method error of about 7%. The C-values < 50 for all the nanoparticles suggests non-porous character of the material. It is possible that significantly larger surface area of G-TeaNPs (compared to B-TeaNPs and R-TeaNPs) may be one of the reasons of enhanced antimicrobial activity.

For a detailed visualization of TeaNPs, we performed a high-resolution transmission electron microscopy (HRTEM) imaging. The imaging was performed with a FEI TECNAI G2 F20S-TWIN microscope. Liquid samples of TeaNPs were placed on the copper meshes and left to get dried. HRTEM imaging allowed to observe the layer of organic matter on the surfaces of AgNPs. The thickness of the organic layer on TeaNPs was estimated by counts, as a mean value from the measurement from 5 different images of the nanoparticles. Surprisingly, we didn't observed any significant differences in the thickness of organic layer on the surface of different TeaNPs – for B-TeaNPs the organic layer was about 2.52 nm thick, for G-TeaNPs – 2.69 nm, and for R-TeaNPs the thickness was about 2.65 nm (**Figure S3)**. Even though the same set of TeaNPs was used for all the analyses, the differences in their sizes, compared to the results obtained with DLS and SEM (**Figure 1A-F),** may be caused by uncontrollable factors, such as the efficacy of sonication for deagglomerating the nanoparticles after the storage.

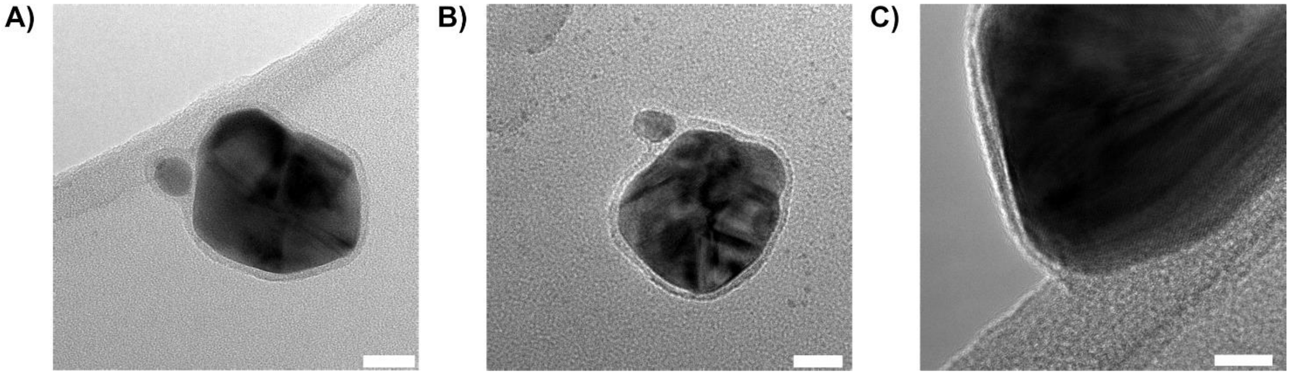

Figure S3. The HRTEM imaging of A) B-TeaNPs, B) G-TeaNPs, C) R-TeaNPs. The scale bar on the images indicates 20 nm (**Figure S3A-B**), or 10 nm (**Figure S3C**).

7. A comparison of antimicrobial activity obtained by using different TeaNPs synthesis methods

Due to a great number of reports describing the synthesis of natural extracts-based AgNPs, we chose three different protocols using tea extracts with minor modifications. Three sets of black, green, and red tea AgNPs, labeled as "1" [1], "2" [2], and "3" [3], were prepared and diluted to reach the concentration of 1 mg/mL. Then, *Escherichia coli* BL21 bacteria were incubated with those nanoparticles according to the protocol described in the *Experimental* chapter of the main manuscript text. Student's t-test was performed to

evaluate whether observed differences, compared to the adequate control, were statistically significant (* p < 0.05; ** p < 0.01; *** p < 0.001). The experiments were conducted in triplicate.

Results are presented in **Figure S4**. Among all three tested sets of nanoparticles, only TeaNPs synthesized using protocol "1" showed significant antibacterial properties. When the survival rate for R-TeaNPs was similar to the control C-AgNPs (about 80%), the incubation in B-TeaNPs and G-TeaNPs for 3 hours resulted in the drop of the survival rate to about 45% and 20% adequately. For TeaNPs synthesized using protocols "2" and "3", the survival rate of *E. coli* bacteria was nonsignificant or comparable to the effects of C-AgNPs.

With this experiment, we proved that the synthesis protocol might affect the antimicrobial effects of obtained TeaNPs, and, therefore, the range of their potential applications. This fact doesn't neglect the usability of TeaNPs synthesized with different protocols for purposes other than microbes elimination.

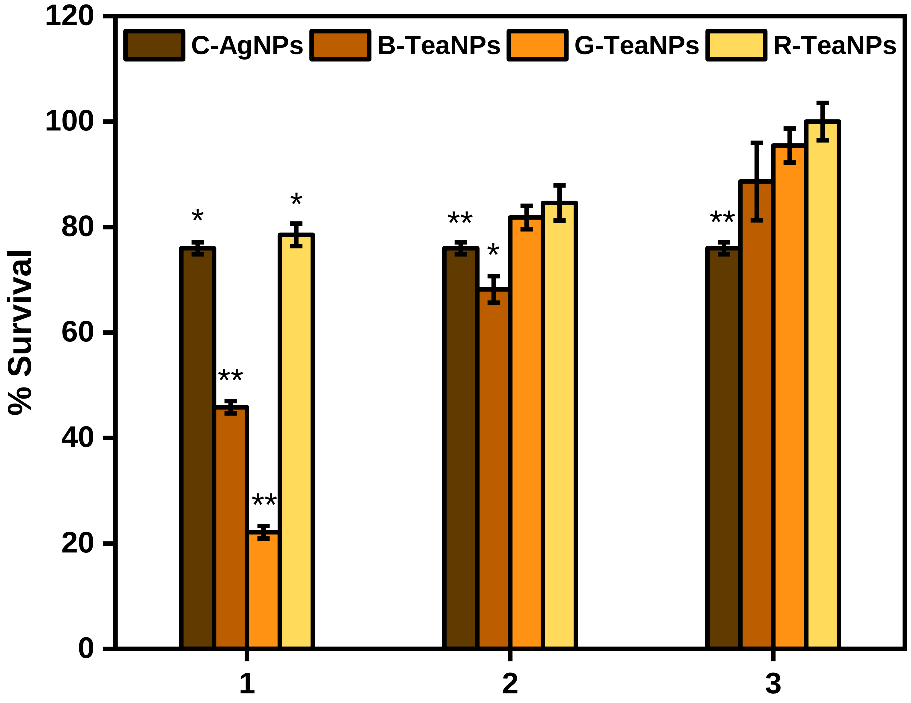

Figure S4. Comparative study of TeaNPs synthesized using three different protocols against *Escherichia coli* BL21. The methods are derived from previously published research, labeled here as 1 [1], 2 [2], and 3 [3]. The results were presented as a percentage of survival (i.e., $\frac{\frac{\mathrm{CFU}}{\mathrm{mL}}\text{ upon exposure}}{\frac{\mathrm{CFU}}{\mathrm{mL}}\text{ in control}}$ * 100%), * p < 0.05; ** p < 0.01; *** p < 0.001, p values were calculated with respect to the control sample (not exposed to any AgNPs).

Additionally, to investigate if the addition of tea extract during the synthesis of AgNPs is crucial to enhance their antimicrobial activity, we performed an experiment in which citrate-capped silver

nanoparticles (C-AgNPs) were mixed with the adequate tea extracts (BT, GT, RT) after the synthesis. In short, we mixed 500 µL of C-AgNPs suspension (1 mg/mL) with 500 µL of adequate tea extract solutions (1 mg/mL; the extracts were first dried, and then resuspended in PBS buffer solution). In the experiment, the mixture was diluted in PBS buffer solution to reach the initial concentration of 0.1 mg/mL. As a control, C-AgNPs suspension was used, along with B-TeaNPs, G-TeaNPs, and R-TeaNPs (0.1 mg/mL). As a model organism, we used *E. coli* BL21 in the initial concentration of about 10^5 CFU/mL. After adding the bacteria to the examined mixtures, all the samples were incubated according to the protocol described in the *Experimental* chapter of the main manuscript text. Student's t-test was performed to evaluate whether observed differences, compared to the adequate control, were statistically significant (* $p < 0.05$; ** $p < 0.01$; *** $p < 0.001$). The experiment was conducted in triplicate.

The results are presented in **Figure S5**. The addition of tea extracts to C-AgNPs enhanced their antibacterial activity, reducing the survival of *E. coli* from 80% to about 60-65%. Minor differences between the samples containing different tea extracts were observed – C-AgNPs with green tea extract (GT) performed slightly better when compared to C-AgNPs with BT and RT (60% survival vs 65% survival). However, with the exception to R-TeaNPs, the mixtures of C-AgNPs with tea extracts performed significantly worse when compared adequate TeaNPs (65% survival in C-Ag+BT and 45% survival in B-TeaNPs; 60% survival in C-Ag+GT and 35% survival in G-TeaNPs).

This experiment proves that the addition of tea extract to chemically synthesized silver nanoparticles (C-AgNPs) can enhance their antibacterial activity, however significantly higher efficacy can be obtained by using the tea extracts during the synthesis of silver nanoparticles (TeaNPs). The antibacterial activity of TeaNPs appeared to be beyond just the additive effect of tea extracts and AgNPs. It is possible that during the synthesis of AgNPs with natural extracts some additional phenomena, that require further studies to be understood, may occur.

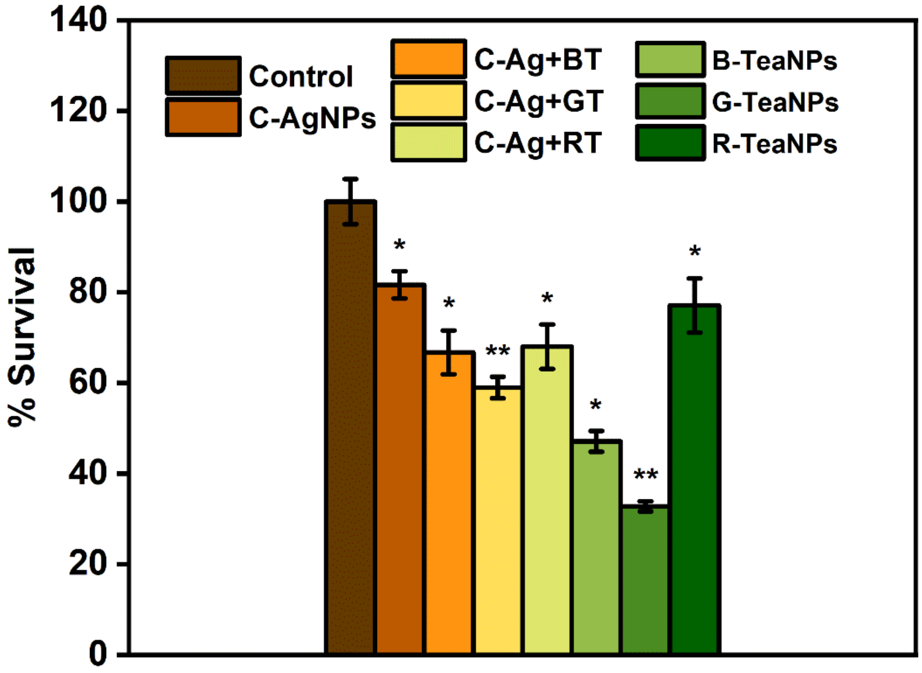

Figure S5. The Comparison of antibacterial activity of citrate-capped silver nanoparticles (C-AgNPs), C-AgNPs mixed with tea extracts (BT, GT, RT) and adequate TeaNPs (B-TeaNPs, G-TeaNPs, R-TeaNPs), presented as the percentage of survival (i.e., $\frac{\frac{CFU}{mL}\,upon\ exposure}{\frac{CFU}{mL}\,in\ control}$ * 100%), * $p < 0.05$; ** $p < 0.01$; *** $p < 0.001$, p values were calculated with respect to the control sample (not exposed to any AgNPs).

References

1 M. Nakhjavani, V. Nikkhah, M. M. Sarafraz, S. Shoja and M. Sarafraz, *Heat Mass Transf. und Stoffuebertragung*, 2017, **53**, 3201–3209.

2 Y. Y. Loo, B. W. Chieng, M. Nishibuchi and S. Radu, *Int. J. Nanomedicine*, 2012, **7**, 4263–4267.

3 S. Babu, M. O. Claville and K. Ghebreyessus, *J. Exp. Nanosci.*, 2015, **10**, 1242–1255.

4 A. Zdunek, M. Krysa and M. Szyma, *Food Chem.*, 2022, **393**, 1–9.

5 Q. Sun, X. Cai, J. Li, M. Zheng, Z. Chen and C. Yu, *Colloids Surfaces A Physicochem. Eng. Asp.*, 2014, **444**, 226–231.
